# Impact of Smoking on Response to the First-Line Treatment of Advanced ALK-Positive Non-Small Cell Lung Cancer: A Bayesian Network Meta-Analysis

**DOI:** 10.3389/fphar.2022.881493

**Published:** 2022-05-11

**Authors:** Kehai Lin, Jie Lin, Zhong Huang, Jiding Fu, Qi Yi, Jiazuo Cai, Muhammad Khan, Yawei Yuan, Junguo Bu

**Affiliations:** ^1^ Department of Oncology, Guangdong Second Provincial General Hospital, Guangzhou, China; ^2^ Department of Radiation Oncology, Affiliated Cancer Hospital & Institute of Guangzhou Medical University, State Key Laboratory of Respiratory Diseases, Guangzhou Institute of Respiratory Disease, Guangzhou, China; ^3^ Department of Intensive Care Unit, Affiliated Cancer Hospital & Institute of Guangzhou Medical University, Guangzhou, China

**Keywords:** non-small cell lung cancer, anaplastic lymphoma kinase, tyrosine kinase inhibitors, smoking, progression-free survival, network meta-analysis

## Abstract

**Background:** The impact of smoking on the efficacy of anaplastic lymphoma kinase (ALK)-positive non-small cell lung cancer (NSCLC) treatment is controversial and has not been systematically explored in the first-line setting. We performed a systematic review based on a pairwise meta-analysis and a Bayesian network meta-analysis (NMA) to address this issue.

**Methods:** PubMed, Embase, Web of Science, Cochrane Library, Clinical-Trials.gov, and other resources were searched until 5 January 2022. Progression-free survival (PFS) was considered the main outcome of interest. Randomized controlled trials with smoking status analysis were included. Cochrane Risk of Bias Tool was performed to assess the risk of bias. Random effects models were adopted conservatively in meta-analysis. The NMA was performed in a Bayesian framework using the “gemtc” version 1.0–1 package of R-4.1.2 software.

**Results:** A total of 2,484 patients from nine studies were eligible for this study, with 1,547 never-smokers (62.3%) and 937 smokers (37.7%). In a pairwise meta-analysis, in the overall population, no significant difference was found between never-smokers and smokers. However, in the subgroup analyses based on crizotinib-controlled studies, anaplastic lymphoma kinase tyrosine kinase inhibitors (ALK-TKIs) derived better PFS in the smoking group over the never-smoking group in the Asian population (HR = 0.17, 95%CI = 0.09–0.31 in the smoking group, HR = 0.39, 95%CI = 0.24–0.65 in the never-smoking group, *p* = 0.04, low quality of evidence). In NMA, among never-smokers, lorlatinib ranked the highest for PFS (SUCRA = 96.2%), but no significant superiority was found among the new-generation ALK-TKIs except for ceritinib. In smokers, low-dose alectinib performed best (SUCRA = 95.5%) and also demonstrated a significant superiority over ensartinib (HR = 0.23, 95%CI = 0.08–0.68, very low quality of evidence), brigatinib (HR = 0.38, 95%CI = 0.14–0.99, low quality of evidence), ceritinib (HR = 0.24, 95%CI = 0.09–0.66, low quality of evidence), crizotinib (HR = 0.18, 95%CI = 0.08–0.41, moderate quality of evidence), and chemotherapy (HR = 0.11, 95%CI = 0.05–0.28, low quality of evidence).

**Conclusion:** In general, smoking may not affect the treatment efficacy of advanced ALK-positive NSCLC in the first-line setting. However, alectinib may perform better in the smoking Asian population. Moreover, lorlatinib in never-smokers and low-dose alectinib in smokers could be considered optimal first-line therapy for advanced ALK-positive NSCLC. Acceptable limitations of evidence, such as study risk of bias, inconsistency, and imprecision, were present in this NMA.

## 1 Introduction

Lung cancer, one of the most malignant tumors in both sexes, ranked first in cancer-related deaths and second in newly diagnosed cancer cases worldwide, with percentages of 18.2% and 12.2%, respectively (Cancer today, 2022). Non-small cell lung cancer (NSCLC) accounts for approximately 85% of lung cancer cases (Thai et al., 2021). Oncogenic alterations gradually play an increasingly important role in the development. It is well-known that smoking rates are high, and the role of different smoking statuses varies significantly in carcinogenesis (Bossé and Amos, 2017; Li et al., 2017; Singal et al., 2019; Wang et al., 2021a; Thai et al., 2021), among which EGFR mutations are the most common oncogenic alterations, ranging from 15% in Europe to 62% in Asia in NSCLC of adenocarcinoma histology. EGFR tyrosine kinase inhibitors have performed well in the targeted treatments, extending patients’ median overall survival to more than 38 months by gefitinib or osimertinib alone, and even to more than 50 months when combined with chemotherapy. Interestingly, more and more oncogenic alterations have also been developed into useful treatment strategies in NSCLC, such as ALK, RET, NTRK, and ROS1 (Thai et al., 2021).

Anaplastic lymphoma kinase (ALK) gene translocation, leading to abnormal expression of constitutively active ALK fusion proteins, is a key mechanism for inducing lung tumorigenesis of ALK-positive NSCLC (3%–5% of NSCLCs). It is more common in never- or light-smokers and younger age and is associated with adenocarcinoma histology (Shaw and Engelman, 2013; Thai et al., 2021). During the past decade, ALK tyrosine kinase inhibitors (ALK-TKIs) have demonstrated remarkable efficacy in the treatment of advanced or metastatic NSCLC and now are the standard options in the first-line treatment of advanced ALK-positive NSCLCs instead of chemotherapy. Compared with cytotoxic chemotherapy, the first-generation ALK-TKI crizotinib significantly has extended median progression-free survival (PFS) of advanced NSCLC (around 11 *vs*. 7 months) (Solomon et al., 2014; Wu et al., 2018). Moreover, the PFS has been remarkably prolonged by the next-generation ALK-TKIs, such as ceritinib, alectinib, brigatinib, ensartinib, and lorlatinib (Soria et al., 2017; Zhou et al., 2019; Mok et al., 2020; Nakagawa et al., 2020; Shaw et al., 2020; Camidge et al., 2021; Horn et al., 2021). Undoubtedly, targeted therapy is the preferred treatment of oncogene-driven advanced NSCLC. Although never- or light-smokers account for a much higher proportion of ALK-positive NSCLC, it is of great interests and necessitous to figure out the effect of smoking on ALK-positive NSCLCs treatment efficacy in the first-line therapy.

To date, previous meta-analyses have investigated the correlation between smoking status and the efficacy of advanced NSCLC treatments (Breadner et al., 2020; Li et al., 2020). However, conflict occurs in the benefit of never-smoking, as one previous meta-analysis found that never-smokers tended to benefit from ALK-TKIs compared with cytotoxic chemotherapy, whereas another denoted that there were similar benefits regardless of the smoking status. Meanwhile, a network meta-analysis focusing on the relative efficacy of first-line targeted therapies in advanced ALK-positive NSCLCs has simply highlighted the role of smoking in the subgroup analysis (Wang et al., 2021b). These previous works could be systematically expanded to determine the correlation between smoking status and efficacy of ALK-targeted agents in the first-line treatment of advanced ALK-positive NSCLC.

Therefore, our study attempted to compare the impact of smoking on the efficacy of advanced ALK-positive NSCLC with high-quality first-line setting randomized controlled trials. Furthermore, a comprehensive NMA of the relative efficacy of first-line treatments according to different smoking statuses was also performed.

## 2 Materials and Methods

This study was carried out following the guidelines of the 2020 Preferred Reporting Items for Systematic Reviews and Meta-Analysis (PRISMA) (Page et al., 2021) and the extension statement of NMA (Hutton et al., 2015). It was registered on the INPLASY website (registration number: INPLASY202180009, https://inplasy.com/inplasy-2021-8-0009/, accessed on 03 August 2021).

### 2.1 Literature Search and Study Selection

We searched PubMed, Embase, Web of Science, Cochrane Library, and Clinical-Trials.gov up to 19 August 2021 without language limitations for eligible studies, which was finally updated on 05 January 2022. In addition, abstracts were searched from the main oncology congresses databases, including the American Society of Clinical Oncology (ASCO), the European Society for Medical Oncology (ESMO), and the World Conference on Lung Cancer (WCLC). The search strategy is presented in Supplementary Table S2. The following search terms were used: non-small cell lung cancer (NSCLC), anaplastic lymphoma kinase (ALK), ALK tyrosine kinase inhibitors, crizotinib, ceritinib, alectinib, brigatinib, ensartinib, lorlatinib, entrectinib, and their medical subject headings (MeSH) terms. The inclusion criteria were as follows: 1) randomized controlled trials with clinical outcomes, such as PFS and OS; 2) patients with pathologically confirmed locally advanced or metastatic NSCLC; 3) studies with clear baseline characteristics of patients and ALK mutation status; and 4) studies including data of smoking status analysis required for meta-analysis. The relevant titles and abstracts were screened to remove duplicated and irrelevant publications. Then, the full texts and relevant reference lists of the other articles were browsed thoroughly for the final inclusion.

### 2.2 Data Extraction and Quality Assessment

The following information was extracted: the trial name, publication year, design, interventions, sample size, race, patients age and gender, baseline brain metastases, adverse effects, previous treatments, number of smokers (defined as current and/or former smokers) and never-smokers, and hazard ratio (HR) with 95% confidence intervals (CIs) for PFS of whole group and subgroup. Quality assessment was performed using the Cochrane Risk of Bias Tool (Higgins et al., 2011). It includes seven domains (random sequence generation, allocation concealment, blinding of participants and personnel, blinding of outcome assessment, incomplete outcome data, selective outcome reporting, and other bias), for which a judgment (low, high, or unclear risk) was assessed respectively. Study selection, data extraction, and risk of bias assessment were independently executed by two reviewers (KL, JL). For any unresolved discrepancies, a third reviewer (ZH) was concerned.

### 2.3 Data Synthesis and Statistical Analysis

PFS was defined as the time from randomization to RECIST-defined disease progression or death from any cause. The HR was regarded as a measure of effect size for PFS. Because overall survival (OS) is immature for most trials and there is a lack of smoking subgroup analysis results, this part of the analysis was not performed.

#### 2.3.1 Pairwise Meta-Analysis

PFS-HR of current smokers and former smokers was combined as the smoker group when smoking statuses were multiply categorized. The HR of current smokers in some studies was ignored because it was not applicable due to a small population. Heterogeneity across studies was assessed by I^2^ statistics, with I^2^ < 25%, 25% ≤ I^2^ ≤ 50%, and I^2^ > 50% being interpreted as signifying low-level, intermediate-level, and high-level heterogeneity, respectively. If necessary, subgroup analysis would be performed. Any heterogeneity between the smoker subgroup and never-smoker subgroup was detected by the Cochran Q test. A *p*-value (two-sided) of less than 0.05 was considered statistically significant. The analysis process was carried out by RevMan 5.4.1, applying the random-effect model conservatively.

#### 2.3.2 Network Meta-Analysis

With the model of the lower deviance information criterion (DIC), which is more feasible (Oravecz and Muth, 2017), a network meta-analysis of different therapeutic drugs in both never smokers and smokers based on a Bayesian framework was performed using the “gemtc 1.0–1” package of R software (version 4.1.2) (Neupane et al., 2014; Tonin et al., 2017). This method integrated both direct and indirect comparisons for any given pair of managements and certain endpoints. The function mtc.run was applied to generate samples, and we set 10000 simulations for each chain as the “burn-in” period, yielding 50,000 iterations to obtain the HR of model parameters when four Markov chains run simultaneously. The Brooks–Gelman–Rubin diagnosis plots method, trace plot, and density plot were used to access the model convergence (Wu et al., 2013). Rank probabilities were calculated to obtain the hierarchy of each treatment, and a plot of rank probabilities was created by the “gemtc” package (Gelman and Rubin, 1992). The probability of the competing treatments was ranked by the surface under the cumulative ranking curve (SUCRA), the highest and lowest values of which mean the highest probability of ranking the best and worst, respectively (Salanti et al., 2011; Tonin et al., 2017).

Stata/SE 15.1 and RevMan 5.4.1 were used to generate network and funnel plots for a visual illustration of relationships among each treatment and evaluation of the studies' publication bias. The mtc.anohe command of the “gemtc” package was used to evaluate global heterogeneity. A sensitivity analysis was performed by removing the trials deemed to be heterogeneous to ensure reliability.

### 2.4 Quality of Evidence

The quality of evidence was assessed in accordance with the GRADE working group approach (Guyatt et al., 2008; Puhan et al., 2014). In this method, the quality of evidence was categorized into four levels (high, moderate, low, and very low), and the starting point of quality of direct evidence based on RCTs would be high, which could be downrated to moderate, low, or very low according to five domains (risk of bias, indirectness, imprecision, inconsistency, and publication bias). We used the GRADEpro Guideline Development Tool (www.gradepro.org) to rate the quality of evidence in a pairwise meta-analysis. In network meta-analysis (Puhan et al., 2014), the quality of indirect evidence was consistent with the lower confidence rating of two direct comparisons that contribute as first-order loops to the indirect estimates. As there are no closed loops in this NMA, the direct or indirect estimates constituted the final outcome.

## 3 Results

### 3.1 Literature Search

**FIGURE 1:**
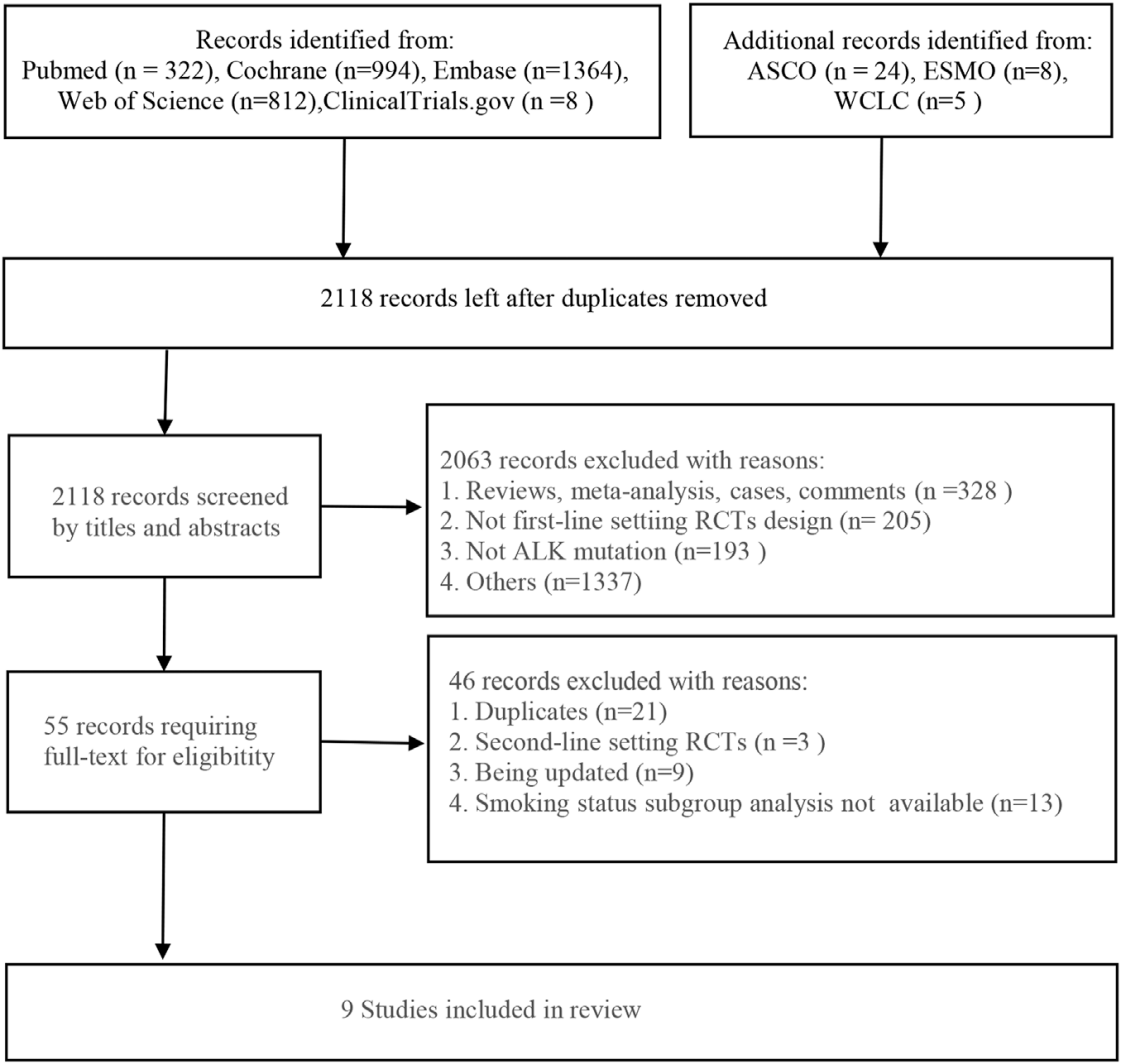
Flowchart of the literature screening. ASCO, the American Society of Clinical Oncology; ESMO, the European Society for Medical Oncology; WCLC, the World Conference on Lung Cancer; RCTs, randomized controlled trials; ALK, anaplastic lymphoma kinase.

Initially, 3,537 eligible studies were yielded from the searching strategies, including 322 studies from PubMed, 1,364 studies from Embase, 812 from Web of Science, 994 studies from Cochrane Library, and 8 studies from Clinical-Trials.gov. In addition, 37 studies were found from other sources. After removing 1,419 duplicates, additional 2,063 studies were excluded by screening the title and abstract. Eventually, nine studies were included in our analysis according to the inclusion criteria. The flowchart of the literature screening is presented in Figure 1.

### 3.2 Study Characteristics

Table 1 presents the detailed characteristics of included studies. All of the included nine studies were phase 3 randomized controlled trials, enrolling 2,484 participants totally, with 1,547 never-smokers (62.3%) and 937 smokers (37.7%). Among them, three were cytotoxic chemotherapy-controlled studies, with PROFILE1014 and PROFILE1029 investigating crizotinib and ASCEND-4 investigating ceritinib and six were crizotinib-controlled studies, with eXalt3 investigating ensartinib, ALTA-1L investigating brigatinib, CROWN investigating lorlatinib, and J-ALEX, ALEX, and ALESIA investigating alectinib). For race differences, three studies were Asian-only trials (PROFILE 1029, J-ALEX, and ALESIA), and six were multi-race trials (Crown, ALEX, ALTA-1L, PROFILE 1014, eXalt3, and ASCEND-4).

**TABLE 1:** Characteristics of the included studies of first-line ALK-TKI treatment for advanced ALK-positive NSCLC.

Study, year	Design	Drug	Sample size	Nonsmoker	Only Asian	PC (%)	Age (median)	Male (%)	BM (%)	G3AE (%)	PFS (months)	HR (95%CI)
PROFILE1014 Solomon et al. (2014)	Phase III, open-label, RCT	Criz	172	106	No	0	52	40	26	NA	10.9 (8.3–13.9)	0.45
2014		Chem	171	112		0	54	37	27	NA	7.0 (6.8–8.2)	(0.35–0.60)
PROFILE1029 Wu et al. (2018)	Phase III, open-label, RCT	Criz	104	78	Yes	0	48	48.1	20.2	NA	11.1 (8.3–12.6)	0.402
2018		Chem	103	72		0	50	41.7	31.1	NA	6.8 (5.7–7.0)	(0.286–0.565)
ASCEND-4 Soria et al. (2017)	Phase III, open-label, RCT	Ceri	189	108	No	0	55	46	31	NA	16·6 (12·6–27·2)	0·55
2017		Chem	187	122		0	54	39	33	NA	8·1 (5·8–11·1)	(0·42–0·73)
J-ALEX Nakagawa et al. (2020)	Phase III, open-label, RCT	Alec_L	103	56	Yes	36	61	40	14	36.9	34.1 (22.1–NR)	0·37
2020		Criz	104	61		36	59.5	39	28	60.6	10·2 (8·3–12·0)	(0.26–0.52)
ALESIA Zhou et al. (2019)	Phase III, open-label, RCT	Alec_H	125	84	Yes	0	51	51	35	29	NR (20·3–NR)	0·22
2019		Criz	62	45		0	49	55	37	48	11·1 (9·1–13·0)	(0·13–0·38)
ALEX Mok et al. (2020)	Phase III, open-label, RCT	Alec_H	152	92	No	0	58	45	42	52	34.8 (17.7–NR)	0.43
2020		Criz	151	98		0	54	42	38	56.3	10.9 (9.1–12.9)	(0.32–0.58)
ALTA-1L Camidge et al. (2021)	Phase III, open-label, RCT	Brig	137	84	No	26	58	50	29	78	24.0 (18.5–43.2)	0.48
2021		Criz	138	75		27	60	41	30	64	11.1 (9.1–13)	(0.35–0.66)
CROWN Shaw et al. (2020)	Phase III, open-label, RCT	Lorl	149	81	No	0	61	44	26	77.2	NR (NR–NR)	0.28
2020		Criz	147	94		0	56	38	27	60.6	9.3 (7.6–11.1)	(0.19–0.41)
eXalt3 Horn et al. (2021)	Phase III, open-label, RCT	Ensa	143	85	No	23.8	54	50.3	32.9	50.4	25.8 (21.8-NR)	0.51
2021		Criz	147	94		28.6	53	52.4	38.8	42.4	12.7 (9.2–16.6)	(0.35–0.72)

ALK, anaplastic lymphoma kinase; TKI, tyrosine kinase inhibitor; NSCLC, non-small cell lung cancer; RCT, randomized controlled trial; Chem, chemotherapy (cisplatin [75 mg/m2], or carboplatin [target area under the curve of 5–6] plus pemetrexed [500 mg/m2]) given every 21 days; Criz, crizotinib (250 mg twice daily); Brig, brigatinib (90 mg once daily for 7 days, then 180 mg once daily); Ceri, ceritinib (750 mg once daily); Ensa, ensartinib (225 mg once daily); Alec_L, low-dose alectinib (300 mg twice daily); Alec_H, high-dose alectinib (600 mg twice daily); Lorl, lorlatinib (100 mg once daily); PC, previous chemotherapy; BM, brain metastasis; G3AE, adverse event ≥ grade 3; HR, hazard ratio; CI, confidence interval; NA, not available; NR, not reached.

### 3.3 Quality Evaluation

All nine studies were open-label studies prone to a high risk of performance bias. However, a low risk of detection bias was observed in all included studies due to the blinding of outcome assessment completed by a blinded independent review committee. Unclear risk occurred in selection bias, attrition bias, reporting bias, and other bias due to the lack of detailed information. Detailed quality assessment is illustrated in Supplementary Figure S1.

Both the trace and density plot (Supplementary Figure S2 for never-smoker, Supplementary Figure S3 for smoker) and Brooks–Gelman–Rubin diagnosis plot (Supplementary Figure S2 for never-smoker, Supplementary Figure S3 for smoker), showing no single chain fluctuation and normal distribution of density map, illustrated an excellent convergence of the models performed in NMA. As seen in Supplementary Figure S4, there was no significant publication bias in the pooled analyses. In terms of inconsistency analyses, the global analysis showed low heterogeneity in nonsmokers and moderate heterogeneity in the smokers (I^2^ = 4%, I^2^ = 26%, respectively). As no closed loop exits in this NMA, local inconsistency analysis was not performed. Thus, a satisfactory consistency among the studies was obtained. The transitivity across the included studies was well balanced by strictly including RCTs according to the selection criteria in this NMA.

### 3.4 Pooled Efficacy for Never-Smokers *Versus* Smokers

**FIGURE 2:**
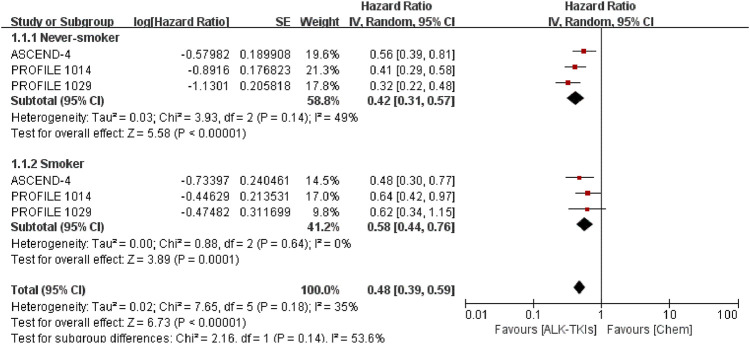
Comparison between smoking status subgroups in chemotherapy-controlled studies. ALK-TKIs, anaplastic lymphoma kinase tyrosine kinase inhibitors; Chem, chemotherapy.

We compared the pooled efficacy for never-smokers against smokers in the chemotherapy-controlled studies and crizotinib-controlled studies, respectively. In chemotherapy-controlled studies (Figure 2, Supplementary Table S3), the pooled PFS-HR for never-smokers was 0.42 (95%CI = 0.31–0.57, moderate quality of evidence), while 0.58 (95%CI = 0.44–0.76, moderate quality of evidence) for smokers. Compared with chemotherapy, treatment with ALK-TKIs exhibited no statistically significant difference between smokers and never-smokers (*p* = 0.14).

**FIGURE 3:**
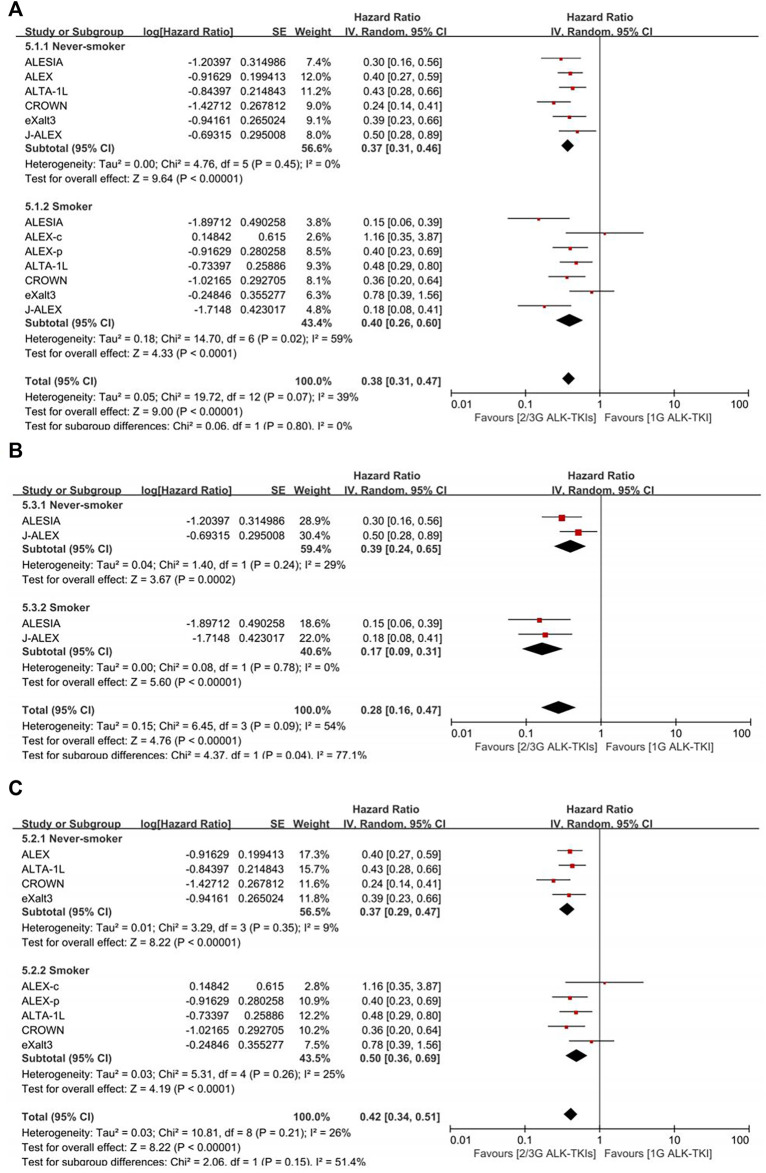
Comparison between smoking status subgroups in crizotinib-controlled studies. **(A)** Initial analysis. **(B)** Asian-only subgroup analysis. **(C)** Multiracial subgroup analysis. ALEX-c, current smoker subgroup of ALEX; ALEX-p, previous smoker subgroup of ALEX; 2/3G ALK-TKIs, both second- and third-generation anaplastic lymphoma kinase tyrosine kinase inhibitors; 1G ALK-TKI, first-generation anaplastic lymphoma kinase tyrosine kinase inhibitor.

In crizotinib-controlled studies (Figure 3, Supplementary Table S4), the pooled PFS-HR analysis yielded 0.37 (95%CI = 0.31–0.46, moderate quality of evidence) for never-smokers and 0.40 (95%CI = 0.26–0.60, very low quality of evidence) for smokers. Compared with crizotinib, treatment with both the second- and third-generation (2/3G) ALK-TKIs presented similar benefits between smokers and never-smokers (*p* = 0.80). The smoker subgroup exhibited significant heterogeneity (I^2^ = 59%). As such, we conducted subgroup analysis by race. In Asian-only studies, pooled PFS-HR was 0.39 (95%CI = 0.24–0.65, moderate quality of evidence) for never smokers and 0.17 (95%CI = 0.09–0.31, low quality of evidence) for smokers, with significant difference (*p* = 0.04). In others, pooled PFS-HR 0.37 (95%CI = 0.29–0.47, moderate quality of evidence) for never smokers and 0.50 (95%CI = 0.36–0.69, moderate quality of evidence) for smokers had no significant difference (*p* = 0.15). Moreover, no significant heterogeneity was found in both subgroup analysis (Asian-only subgroup: I^2^ = 29% in never-smoker subgroup, 0% in smoker subgroup; multirace subgroup: I^2^ = 9% in never-smoker subgroup, 25% in smoker subgroup).

### 3.5 Network Meta-Analysis for Efficacy

**FIGURE 4:**
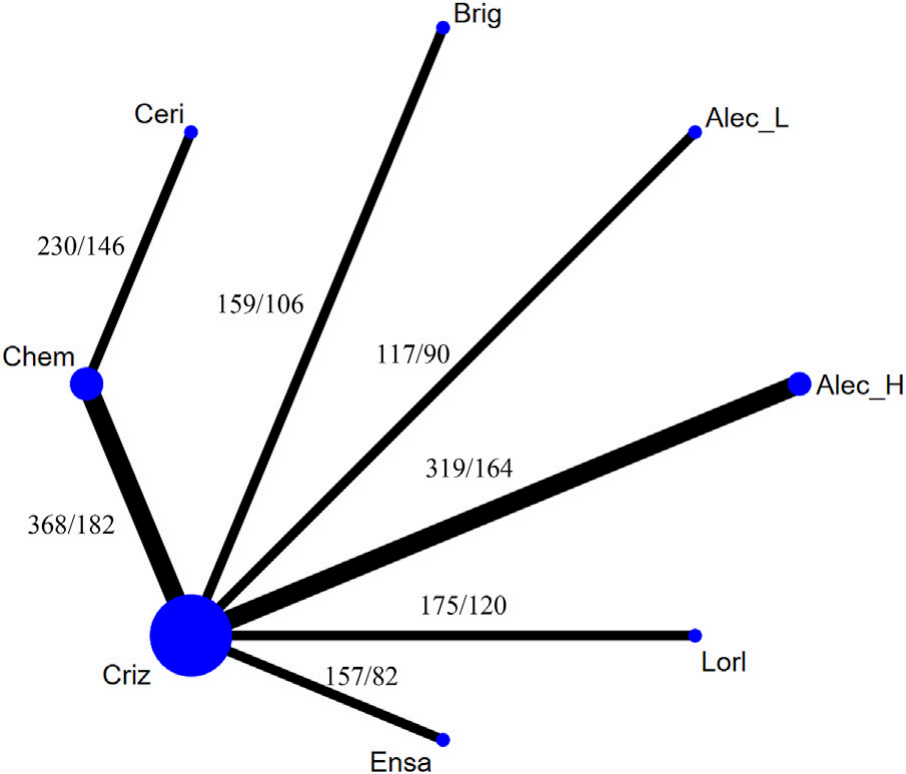
Network constructions for comparisons in progression-free survival of the never-smoker group or smoker group. Chem, chemotherapy; Criz, crizotinib; Brig, brigatinib; Ceri, ceritinib; Ensa, ensartinib; Alec_L, low-dose alectinib; Alec_H, high-dose alectinib; Lorl, lorlatinib. The “number 1/number 2” listed in the upper left of each comparison means that number 1 is the number of never-smokers and number 2 is the number of smokers in each comparison.

Figure 4 presents the network plot of each treatment. Eight treatments were involved in this NMA, with low-dose alectinib (ld-alectinib, 300 mg twice daily) and high-dose alectinib (hd-alectinib, 600 mg twice daily) being regarded as separate treatments.

#### 3.5.1 Network Meta-Analysis for Efficacy in the Never-Smoker Group

**FIGURE 5:**
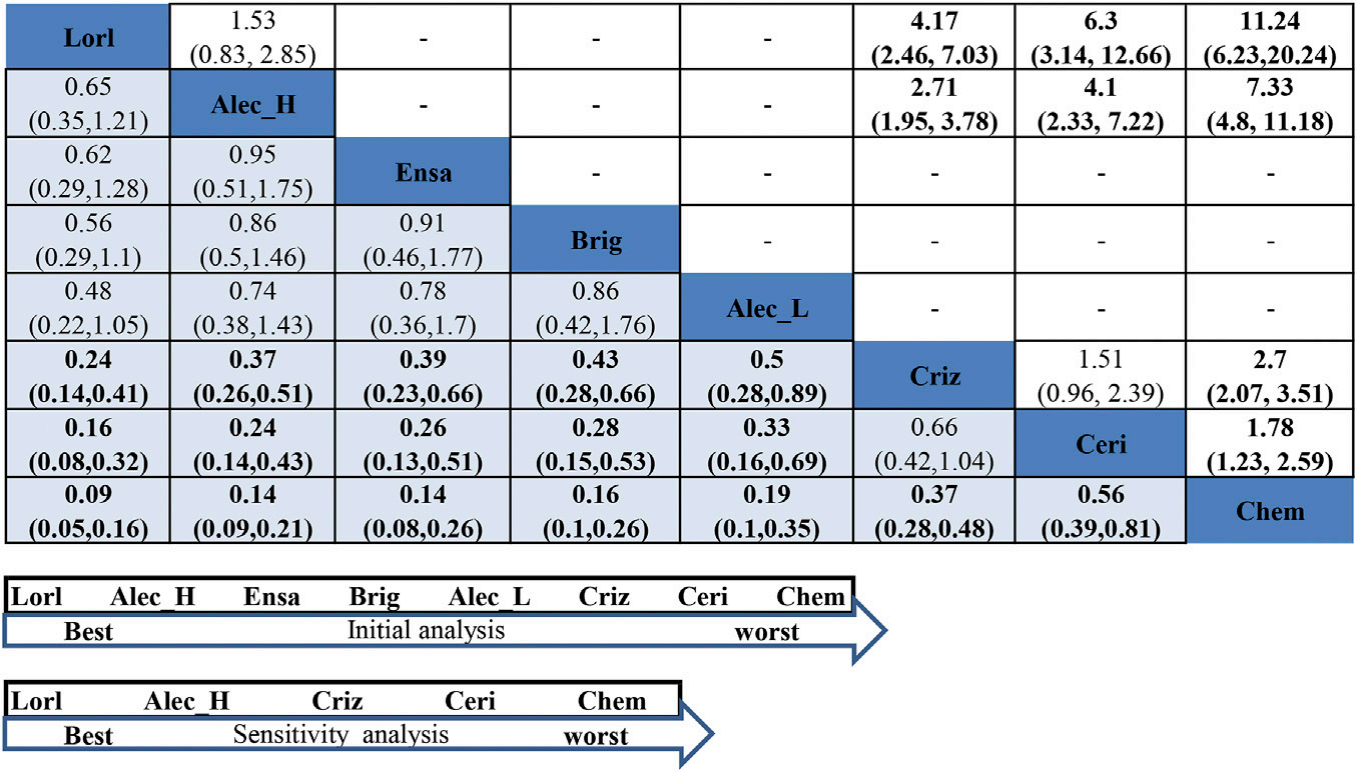
NMA results of never-smoker on progression-free survival (lower left) and sensitivity analysis (upper right), followed by the ranking distribution according to SUCRA values (lower arrow shape). NMA, network meta-analysis; SUCRA, the surface under the cumulative ranking curve; Chem, chemotherapy; Criz, crizotinib; Ceri, ceritinib; Alec_L, low-dose alectinib; Alec_H, high-dose alectinib; Brig, brigatinib; Lorl, lorlatinib; Ensa, ensartinib. Values in bold mean statistically significant.

In the never-smoker group, as presented in Figure 5, all ALK-TKIs were significantly superior to chemotherapy; significant superiority was observed for all next-generation ALK-TKIs other than ceritinib when compared to crizotinib (HR = 0.24, 95%CI = 0.14–0.41, moderate quality of evidence for lorlatinib; HR = 0.37, 95%CI = 0.26–0.51, moderate quality of evidence for hd-alectinib; HR = 0.39, 95%CI = 0.23–0.66, moderate quality of evidence for ensartinib; HR = 0.43, 95%CI = 0.28–0.65, moderate quality of evidence for brigatinib; HR = 0.5, 95%CI = 0.28–0.89, low quality of evidence for ld-alectinib; HR = 1.51, 95%CI = 0.96–2.38, low quality of evidence for ceritinib); and no significant difference was noticed between hd- and ld-alectinib (HR = 0.74, 95%CI = 0.38–1.43, low quality of evidence), with the former showing relatively better efficacy. Additionally, lorlatinib, presenting highest SUCRA and Prbest values (SUCRA = 96.2%, Prbest = 82.2%) (Supplementary Tables S5, S6, Supplementary Figure S5), performed best, followed by hd-alectinib (SUCRA = 74.1%), ensartinib (SUCRA = 69.7%), brigatinib (SUCRA = 62.5%), ld-alectinib (SUCRA = 54.5%), crizotinib (SUCRA = 28.2%), ceritinib (SUCRA = 14.8%), and chemotherapy (SUCRA = 0.00%).

For assessment of between-study heterogeneity (Supplementary Figure S6), we found no significant difference either in the crizotinib-chemotherapy comparison in both PROFILE1014 and PROFILE1029 or the comparison between crizotinib and hd-alectinib in both ALEX and ALESIA, with both showing no heterogeneity (I^2^ = 0.0%). Because three trials (J-ALEX, eXalt3, and ALTA-1L) had also enrolled patients with history of previous chemotherapy, a sensitivity analysis was performed by excluding these trials. Then, rank probabilities by SUCRA values were generated in the remaining studies. Figure 5 and Supplementary Table S6 reveal the same results for the relative ranking of the five remaining treatment groups. Based on these results, we may conclude that a history of previous treatment may not affect the outcomes of our NMA.

#### 3.5.2 Network Meta-Analysis for Efficacy in the Smoker Group

**FIGURE 6:**
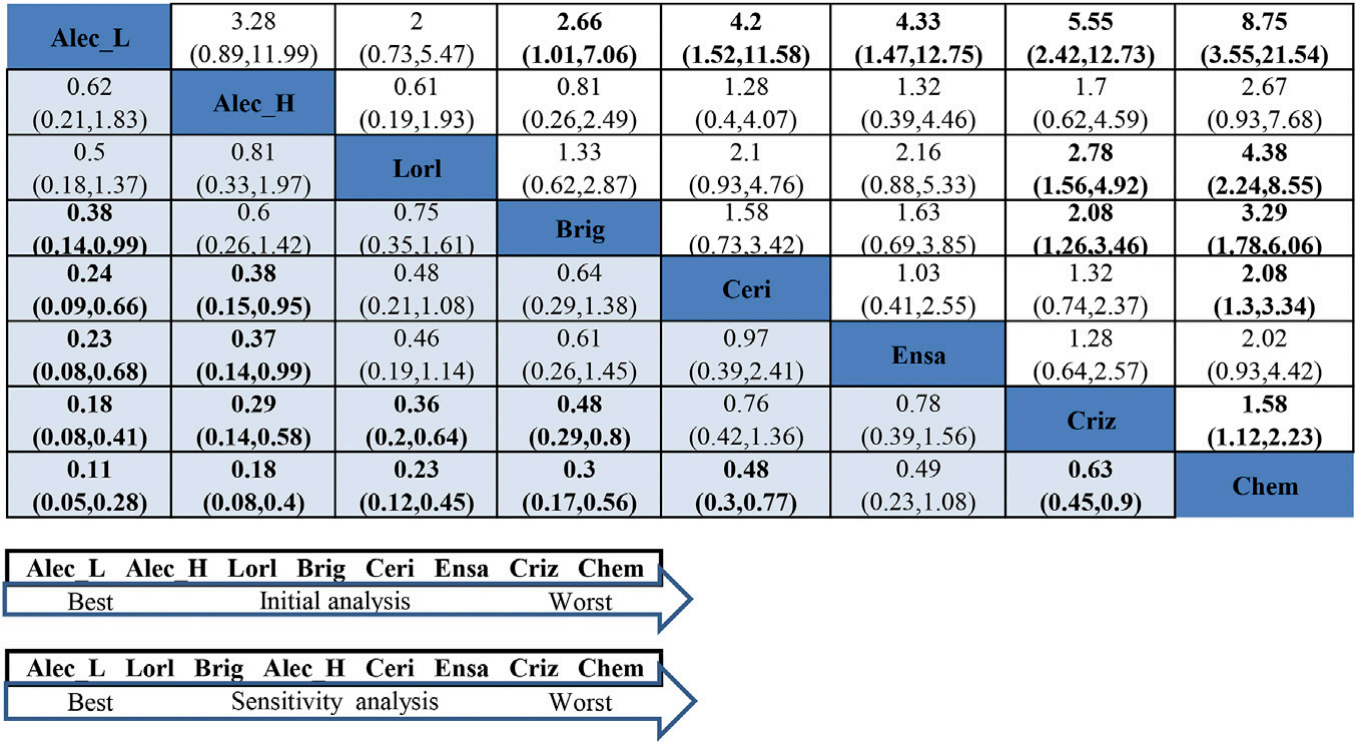
NMA results of smokers on progression-free survival (lower left) and sensitivity analysis (upper right), followed by the ranking distribution according to SUCRA values (lower arrow shape). NMA, network meta-analysis; SUCRA, the surface under the cumulative ranking curve; Chem, chemotherapy; Criz, crizotinib; Ceri, ceritinib; Alec_L, low-dose alectinib; Alec_H, high-dose alectinib; Brig, brigatinib; Lorl, lorlatinib; Ensa, ensartinib. Values in bold mean statistically significant.

In the smoker group, as presented in Figure 6, ld-alectinib showed significantly better PFS than other ALK-TKIs except for hd-alectinib and lorlatinib (HR = 0.18, 95%CI = 0.08–0.41, moderate quality of evidence for crizotinib; HR = 0.23, 95%CI = 0.08–0.68, very low quality of evidence for ensartinib; HR = 0.24, 95%CI = 0.09–0.66, low quality of evidence for ceritinib; HR = 0.38, 95%CI = 0.14–0.99, low quality of evidence for brigatinib; HR = 0.5, 95%CI = 0.18–1.37, moderate quality of evidence for lorlatinib; HR = 0.62, 95%CI = 0.21–1.83, very low quality of evidence for hd-alectinib). Though lorlatinib has demonstrated a greater effect in the never-smoker group, it was not superior to both ld- and hd-alcetinib in PFS for the smoker group (HR = 0.5, 95%CI = 0.18–1.37, moderate quality of evidence for ld-alectinib; HR = 0.81, 95%CI = 0.33–1.97, very low quality of evidence for hd-alectinib). Additionally, ld-alectinib (SUCRA = 95.5%, Prbest = 76.9%) (Supplementary Tables S6, S7 **,** Supplementary Figure S7) was considered to rank first with greatest probability, followed by hd-alectinib (SUCRA = 81.6%), lorlatinib (SUCRA = 72.8%), brigatinib (SUCRA = 58.8%), ceritinib (SUCRA = 36.2%), ensartinib (SUCRA = 34.3%), crizotinib (SUCRA = 20.2%), and chemotherapy (SUCRA = 0.01%).

For assessment of between-study heterogeneity (Supplementary Figure S8), a significant difference was found in the crizotinib-hd-alectinib comparison in both ALEX and ALESIA (I^2^ = 73.5%) but not in the crizotinib-chemotherapy group in both PROFILE1014 and PROFILE1029 (I^2^ = 0). High heterogeneity between ALEX and ALESIA was considered to result from a small number of smokers in the crizotinib arm (only 14) in the ALESIA study. Under such circumstances, we performed a sensitivity analysis by excluding ALESIA. The results (Figure 6 and Supplementary Table S6) showed that ld-alectinib still performed better, and the relative ranking of other treatments was consistent with the result from the initial NMA except for hd-alectinib, which was not better than lorlatinib and brigatinib. More sensitivity analyses by excluding studies enrolling patients with a history of previous chemotherapy are shown in Supplementary Table S6. It is concluded that the similar outcomes, to a certain extent, symbolized the inherent robustness of the NMA, which confirms the ultimate results.

## 4 Discussion

On the one hand, it is well-known that smoking is of great importance in the morbidity and mortality of lung cancer (Loeb et al., 1984; Jung et al., 2016). On the other hand, the impact of smoking on treatment decisions is controversial and is recently being widely investigated (Lin et al., 2018; Li et al., 2020; Xiao et al., 2020; Chen et al., 2021; Zhao et al., 2021). To our knowledge, the comprehensive and systematic analysis of the relationship between smoking status and first-line treatment efficacy of advanced ALK-positive NSCLC has not been reported yet. Our present work may contribute to resolving this discrepancy and provide useful advice for clinical strategy.

As ALK-TKIs have replaced traditional chemotherapies as the upfront treatments with great advantages in efficacy and safety (Guidelines Detail, 2022), we classified the included studies into two groups: the chemotherapy-controlled and crizotinib-controlled groups. Although we found no difference between the smoking group and the never-smoking group in both chemotherapy- and crizotinib-controlled groups, the subgroup analysis by race in the crizotinib-controlled group indicated that ALK-TKIs in Asian-only derived better outcomes in the smoking group compared to the never-smoking group. In contrast to our outcomes, Breadner’s meta-analysis found a greater degree of benefit with ALK-TKIs in the never-smoker group (Breadner et al., 2020). Nonetheless, their study included second-line setting studies in the chemotherapy-controlled group. Moreover, it is possible that the advancement of lung cancer may have constrained the smoking factor from being detected. In real-world data, smoking history has been regarded as an independent negative prognostic factor for survival benefits (Jin et al., 2018; Britschgi et al., 2020). Tobacco use impairs the treatment efficacy of lung cancer and shortens patients’ survival as smoking tobacco directly influences response to anti-tumor drugs by affecting drugs metabolism (Gemine and Lewis, 2016). On the contrary, immunotherapy performs better in NSCLC patients with a smoking history (Li et al., 2020; Nie et al., 2020; Zhao et al., 2021). Notably, both studies in the Asian-only subgroup used alectinib, despite dosage differences, which may suggest the excellent efficacy of alectinib in smokers, while the experimental ALK-TKIs in another group dramatically varied in multiracial studies.

Recently, network meta-analyses have demonstrated the great advantage of both lorlatinib and alectinib on PFS, with the former being the best (Ando et al., 2021; Chuang et al., 2021). Our NMA of the never-smoking group also supports the advantageous effect of lorlatinib on PFS with significant superiority over crizotinib and ceritinib. As it was designed to easily penetrate the blood–brain barrier and against resistance to all known ALK mutants (Johnson et al., 2014; Zou et al., 2015; Solomon et al., 2018; El Darsa et al., 2020), lorlatinib had an extraordinarily good performance on PFS, not only in the frontline therapy but also in subsequent therapy owing to the failure of first- or second-generation ALK inhibitors (Shaw et al., 2020; Kuang and Leighl, 2021). Nevertheless, interestingly, consistent with Chuang’s finding of ld-alectinib ranking first in the patients with baseline brain metastasis (Chuang et al., 2021), the efficacy of ld-alectinib surpassed that of lorlatinib in the NMA of the smoking group, although there were no significant differences. Moreover, ld-alectinib administration resulted in significantly superior PFS to that of ensartinib, ceritinib, crizotinib, and chemotherapy. It is a feature of ld-alectinib, but not hd-alectinib, to show relatively high activity in the smoking subgroup of advanced ALK-positive NSCLC. However, there is little direct evidence to explain the potent mechanism of this discrepancy. To our knowledge, smokers suffer far more mutations than never-smokers, among which TP53 mutation deserves more attention in lung cancer (Le Calvez et al., 2005; Ding et al., 2008). It is noted that there is a high rate of TP53 co-mutation in ALK-positive NSCLC, which has shown a significantly worse prognosis (Aisner et al., 2017; Kron et al., 2018). In Yoda’s research, TP53 mutation coexisted in half of the lorlatinib-resistant samples (Yoda et al., 2018). In the ALTA-1L study (Camidge et al., 2021), patients with TP53 mutant derived apparently shorter PFS not only in the crizotinib treatment group but also in the brigatinib group. However, there was no more information concerning TP53 mutation and efficacy of other ALK-TKIs and relevant correlation with smoking. Probably, the abundant mutations in smokers complicate the drug efficacy. Therefore, additional studies are required to clarify the potential optimal treatment for smokers and never-smokers with elucidation on mechanistic details.

Importantly, adverse effects (AEs) play an indispensable role in clinical treatment decision-making. A lower dose of alectinib, 300 mg twice daily, is a legally experimental dose resulting from Japanese authority due to Japan’s historical maximum intake level of sodium lauryl sulfate, one of the capsule excipients for alectinib (Seto et al., 2013; Zhou et al., 2019). Inconsistent with theoretical assumptions, compared with hd-alectinib, 600 mg twice daily, dose reduction of alectinib did not obviously show a better safety profile in J-ALEX (≥ grade 3 AEs in J-ALEX = 36.9%; ALESIA = 29%; and ALEX = 52%). However, median follow-ups varied in these trials, which may have an impact on the safety profile. Median follow-up was 42.4 months for ld-alectinib in J-ALEX and 48.2 months for hd-alectinib in ALEX, which were much longer than 16.2 months for hd-alectinib in ALESIA (Zhou et al., 2019; Mok et al., 2020; Nakagawa et al., 2020). Also, 36% of participants had received previous chemotherapy in J-ALEX, which may have escalated their adverse effects. Accordingly, the safety profile of ld-alectinib is not worse than that of hd-alectinib, which means ld-alectinib is well tolerated. Lorlatinib (Shaw et al., 2020) is also well tolerated. Although the rate of adverse events (≥ grade 3) was as high as 77%, most of them were hyperlipidemia, weight gain, and hypertension. Moreover, cognitive effects and peripheral neuropathy were common but generally mild, all of which could be well managed.

Lastly, several limitations in our NMA are inevitable. First, in these RCTs, smoking history was not a stratification factor in randomization and small sample sizes existed, probably resulting in heterogeneity in patients selection for our meta-analysis and imprecision of evidence. Second, the direct comparisons were all based on chemotherapy- and crizotinib-controlled studies, which means a lack of direct comparison between next-generation ALK-TKIs and closed loops in the analysis, so the direct evidence among each comparison is insufficient. Third, there is only one trial for each of the next-generation ALK-TKI other than alectinib, and insufficient data may result in instability of the outcome. Fourth, the overall survival of most next-generation ALK-TKIs studies is immature, and subgroup outcome of smoking status is rarely presented. Consequently, extrapolation of long-term outcomes is prevented. Even with the inherent limitations of this NMA, we strictly follow the guidelines of PRISMA and the extension statement of NMA, which improves the quality of our analyses.

## 5 Conclusion

In summary, this systematic review compared the impact of smoking status on treatment efficacy and the relative efficacy of each frontline choice in the first-line setting of advanced ALK-positive NSCLC in terms of PFS. Although, in the overall population, there were no significant differences between smoking statuses, we found in the subgroup analyses that ALK-TKIs derived better PFS in the smoking group over the never-smoking group in the Asian population. Among never-smokers, lorlatinib ranks the highest for PFS, but no significant superiority was found among new-generation ALK-TKIs except for comparison with ceritinib. However, ld-alectinib performed better than lorlatinib among smokers, with ld-alectinib ranking first, followed by lorlatinib, brigatinib, hd-alectinib, ceritinib, ensartinib, crizotinib, and chemotherapy. Moreover, ld-alectinib was significantly superior to ensartinib, brigatinib, ceritinib, crizotinib, and chemotherapy. Given the limitations of this meta-analysis, further research focusing on the smoking status is needed to verify these conclusions.

## Data Availability

The original contributions presented in the study are included in the article/Supplementary Material, further inquiries can be directed to the corresponding authors.
