# Supplementary figures and images for: Impact of Smoking on Response to the First-Line Treatment of Advanced ALK-Positive Non-Small Cell Lung Cancer: A Bayesian Network Meta-Analysis
Source: Front Pharmacol. 2022 May 11;13:881493. doi: 10.3389/fphar.2022.881493 (PMC9130699; doi:10.3389/fphar.2022.881493)

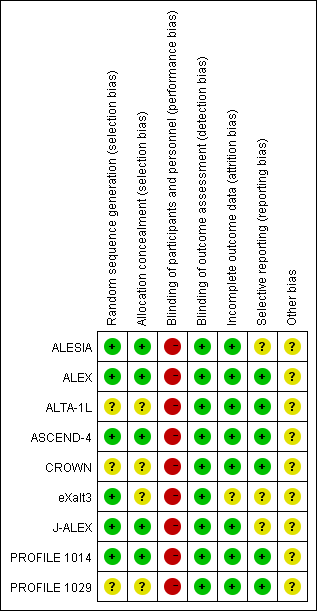


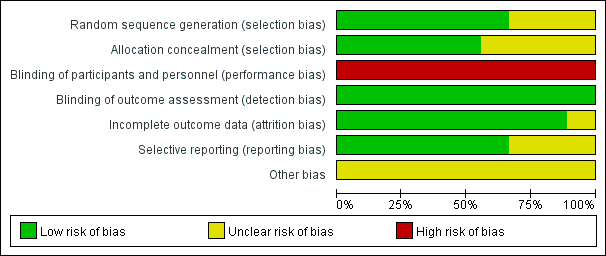

Supplement: Supplementary file 1 [file Table1.DOCX]

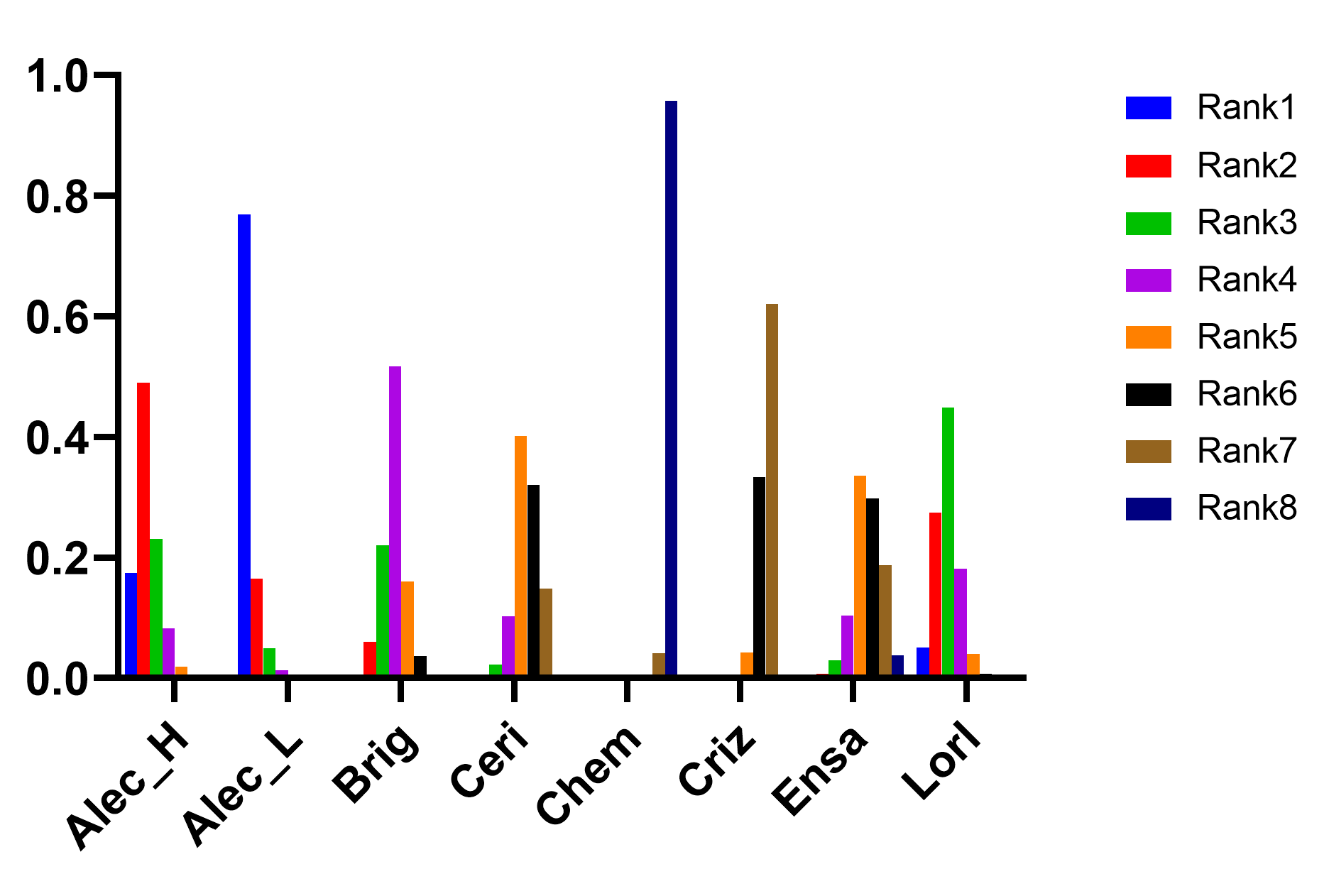

Supplement: Supplementary file 4 [file Image2.TIF]

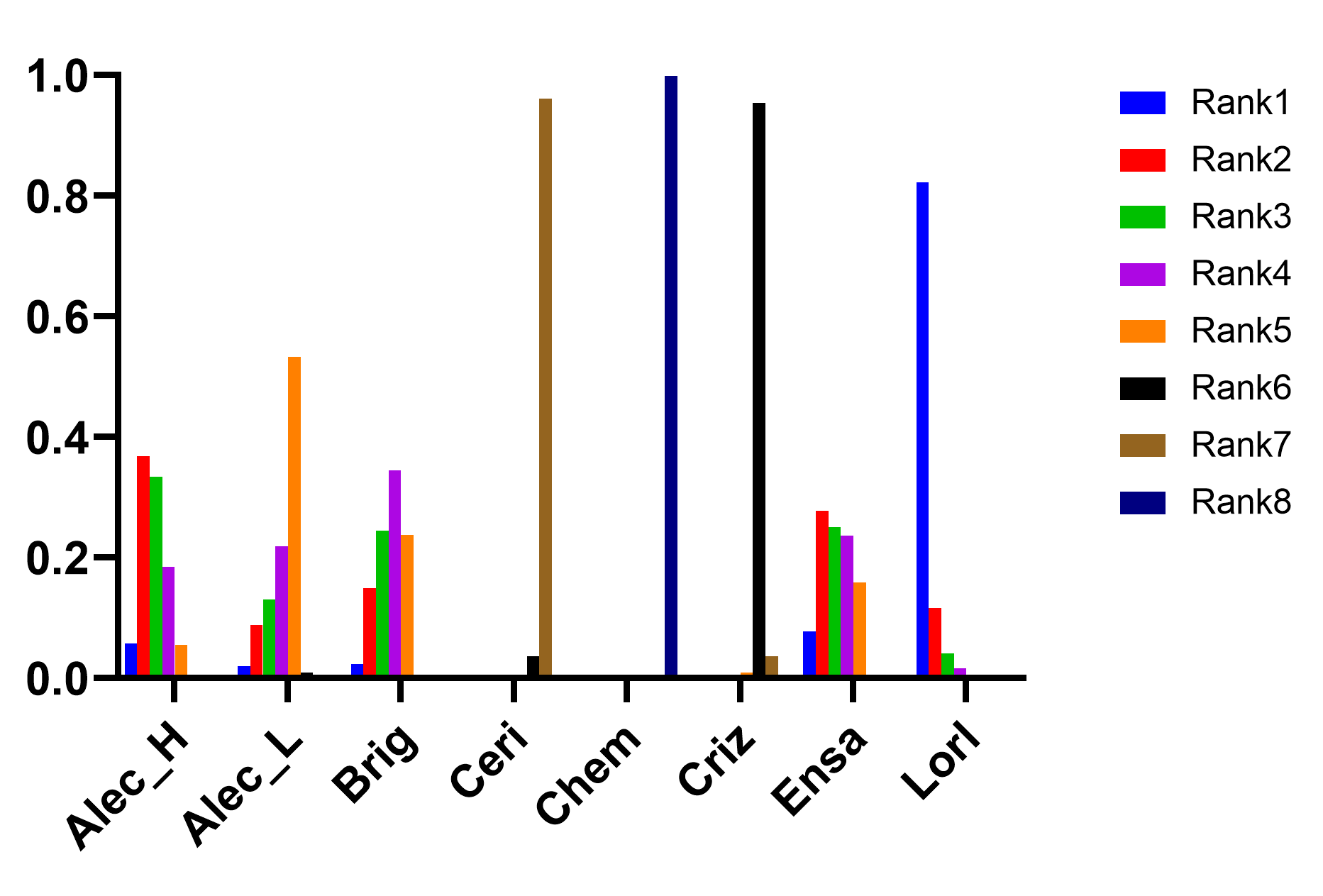

Supplement: Supplementary file 5 [file Image1.TIF]

**(A)**

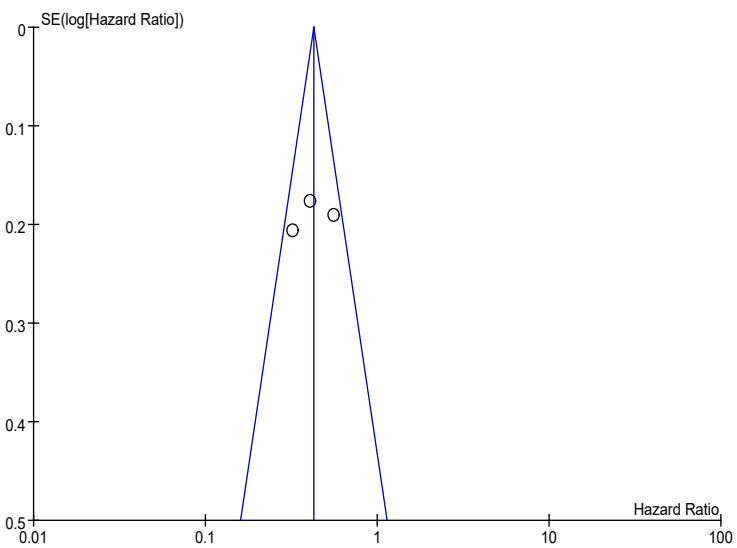

**(B)**

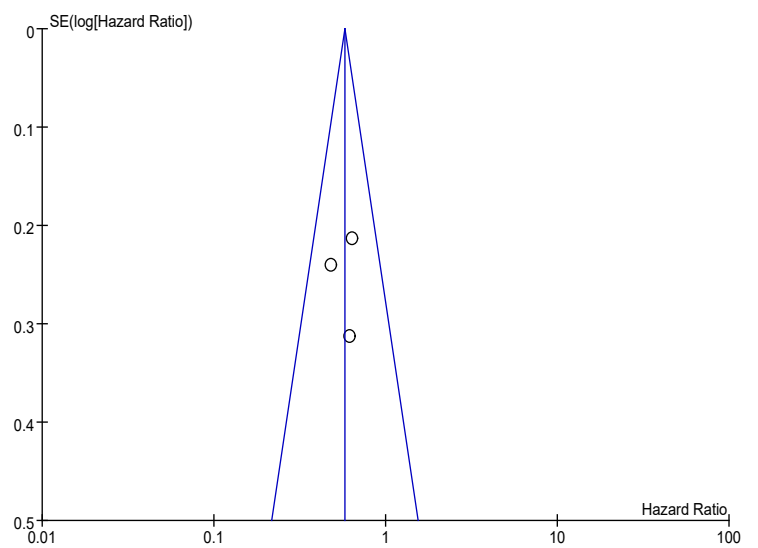

**(C)**

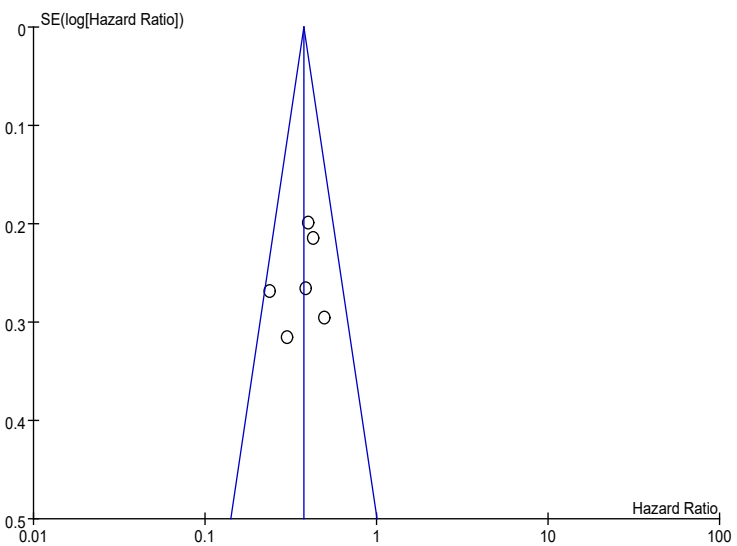

**(D)**

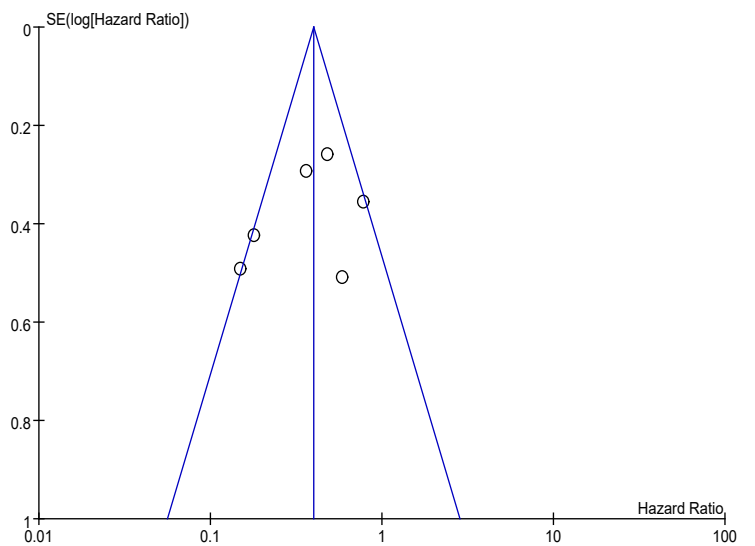

**(E)**

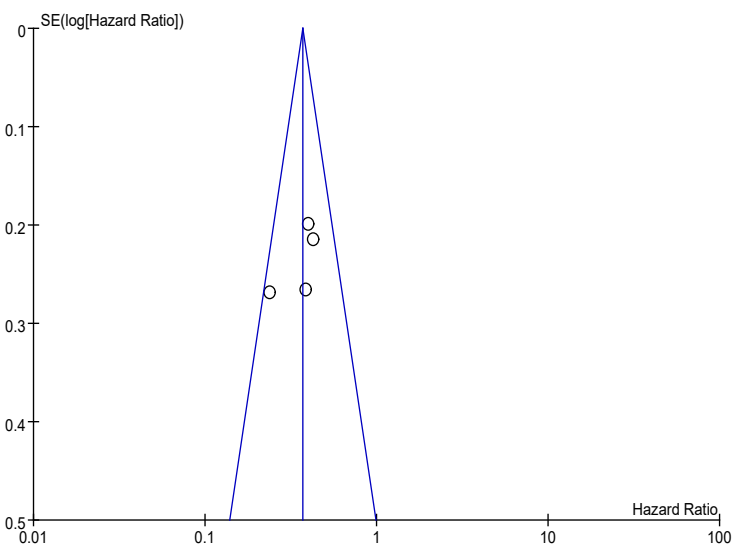

**(F)**

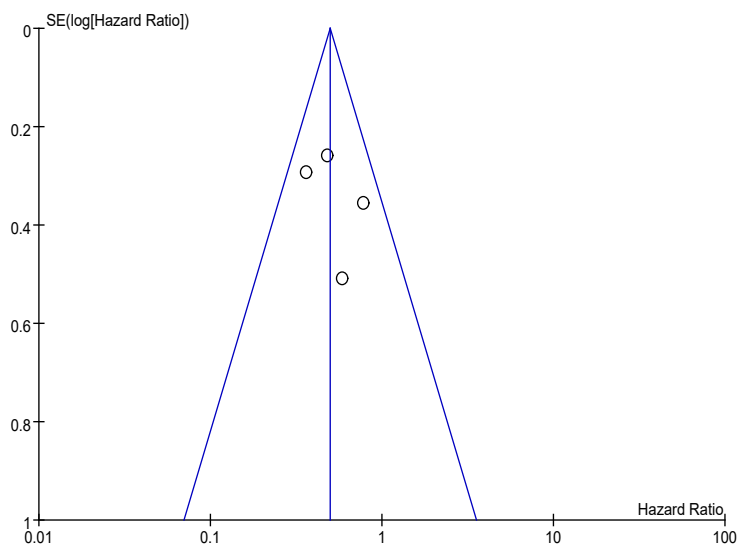

(G)

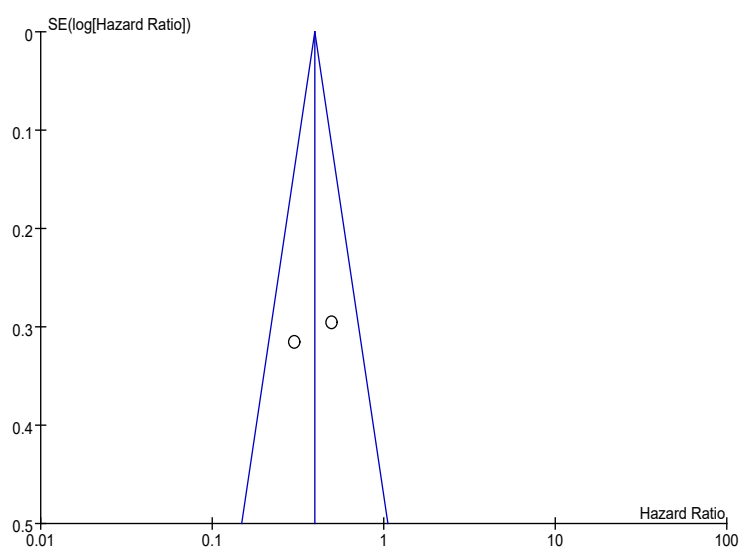

(H)

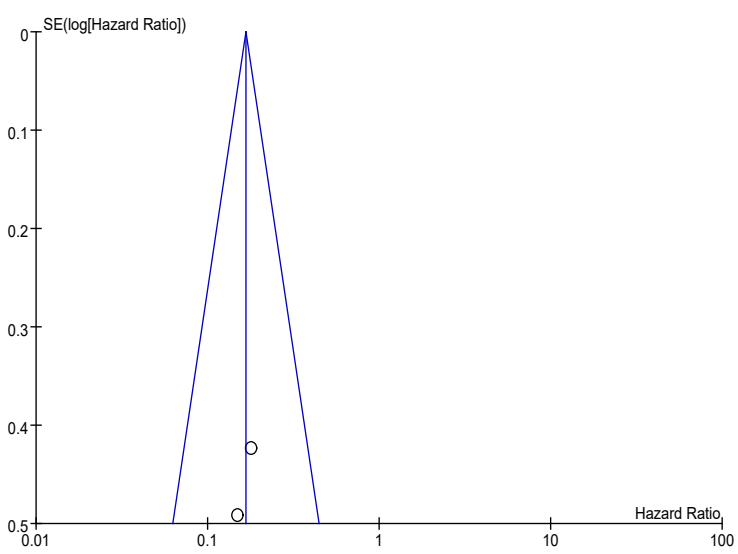

(I)

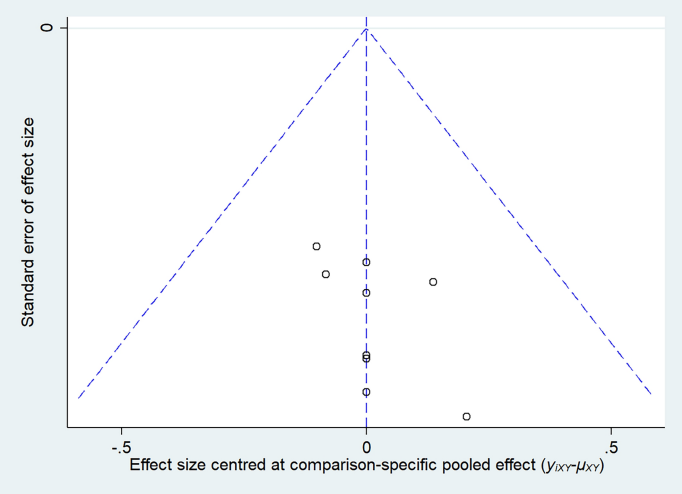

(J)

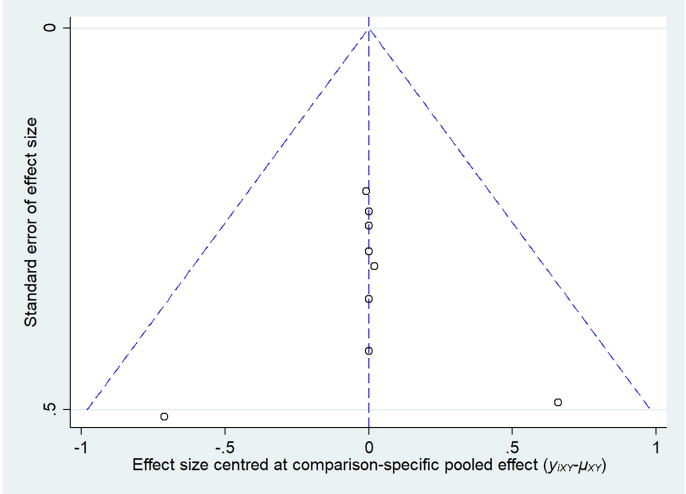

Supplement: Supplementary file 6 [file Image4.pdf]

(A)

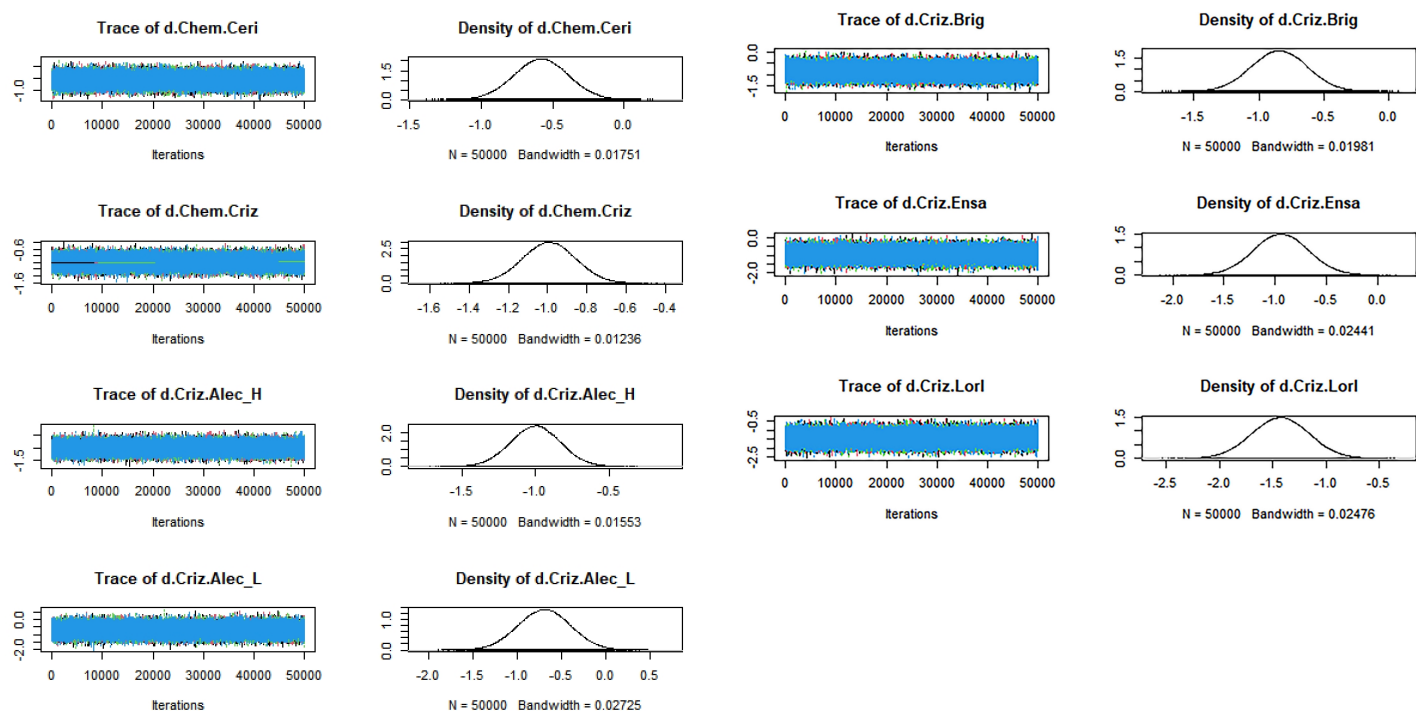

(B)

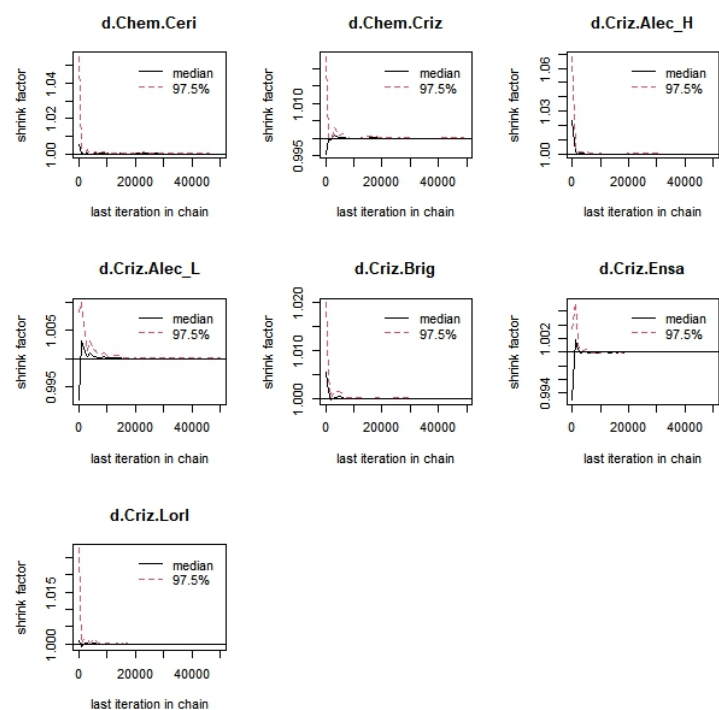

Supplement: Supplementary file 8 [file Image3.pdf]

**(A)**


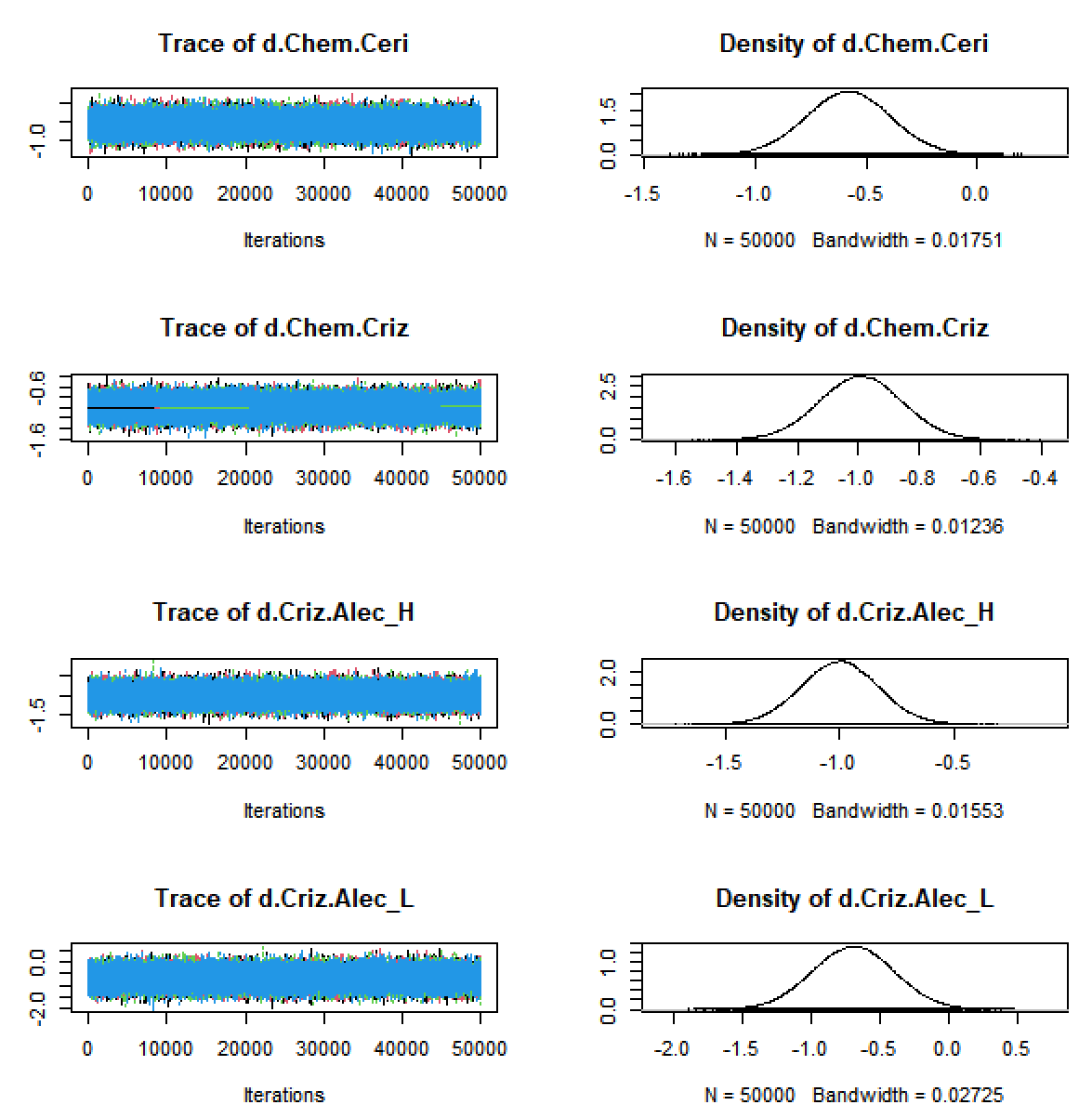

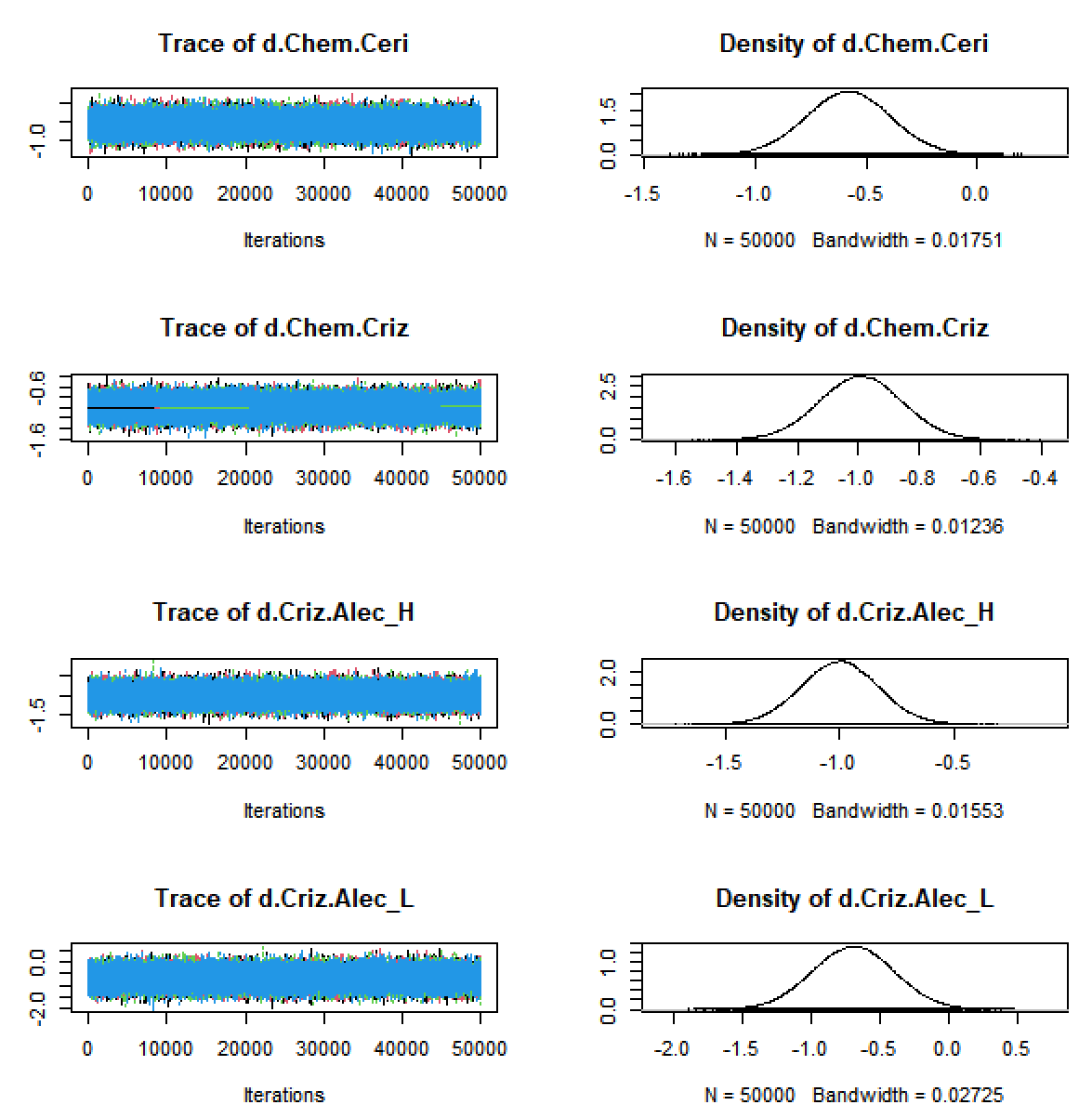


**(B)**


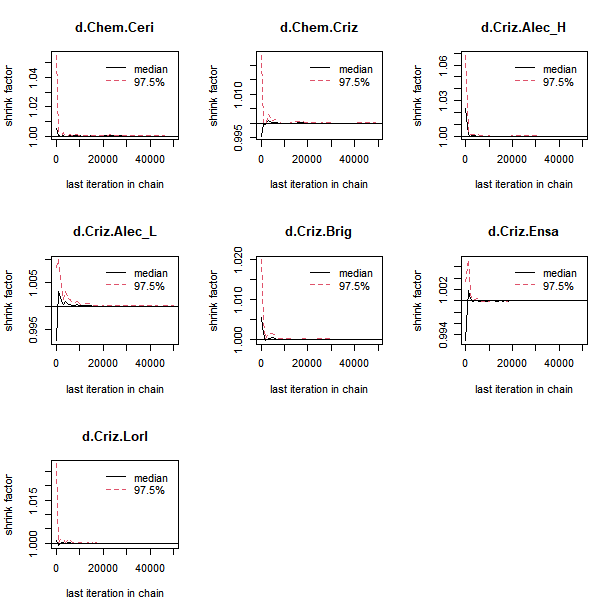

Supplement: Supplementary file 9 [file Table2.DOCX]

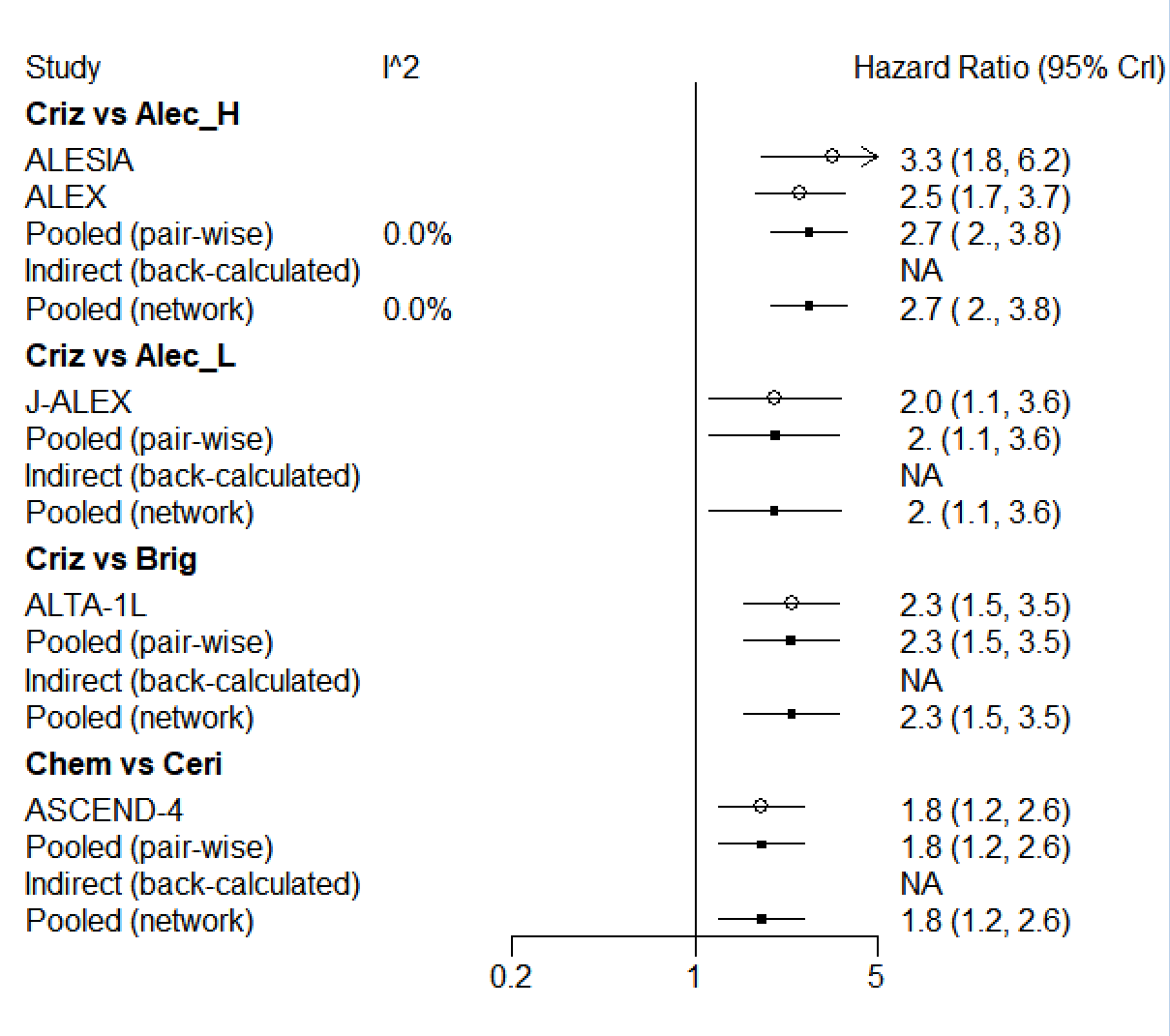

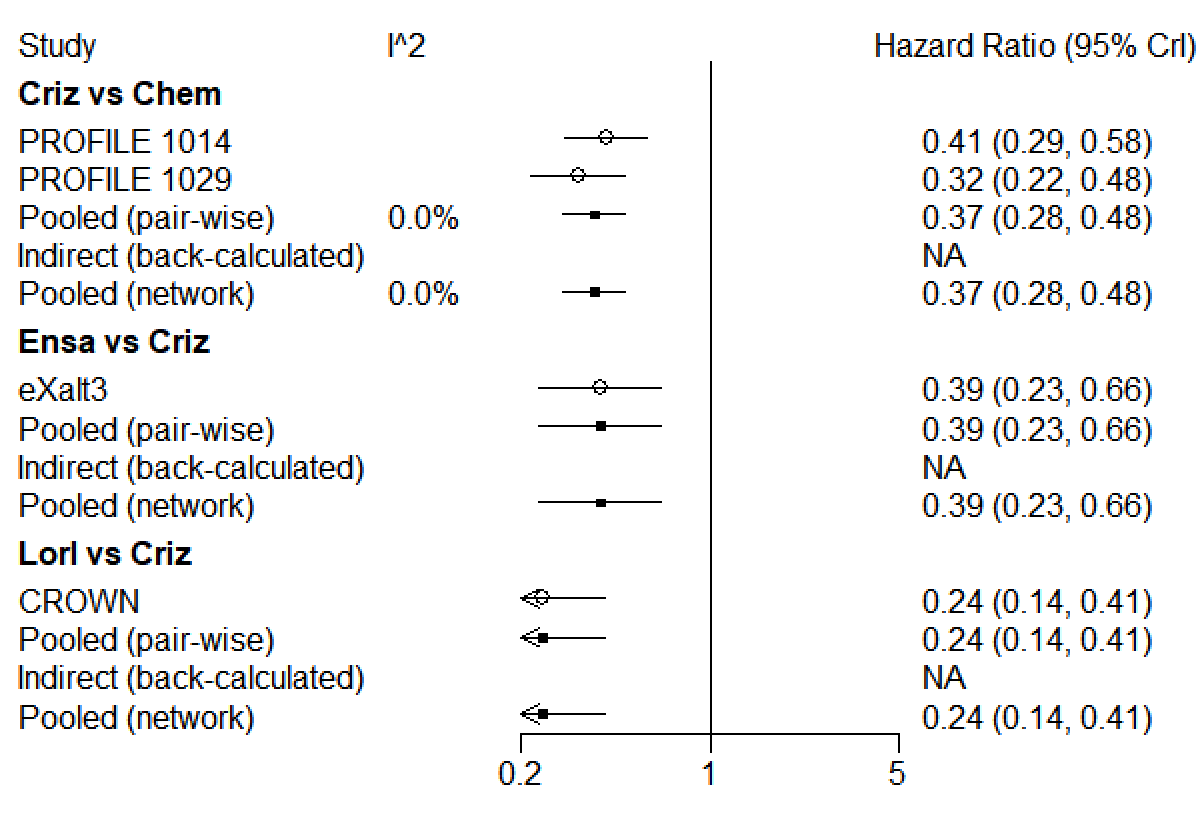

Supplement: Supplementary file 10 [file Table5.DOCX]

**(A)**


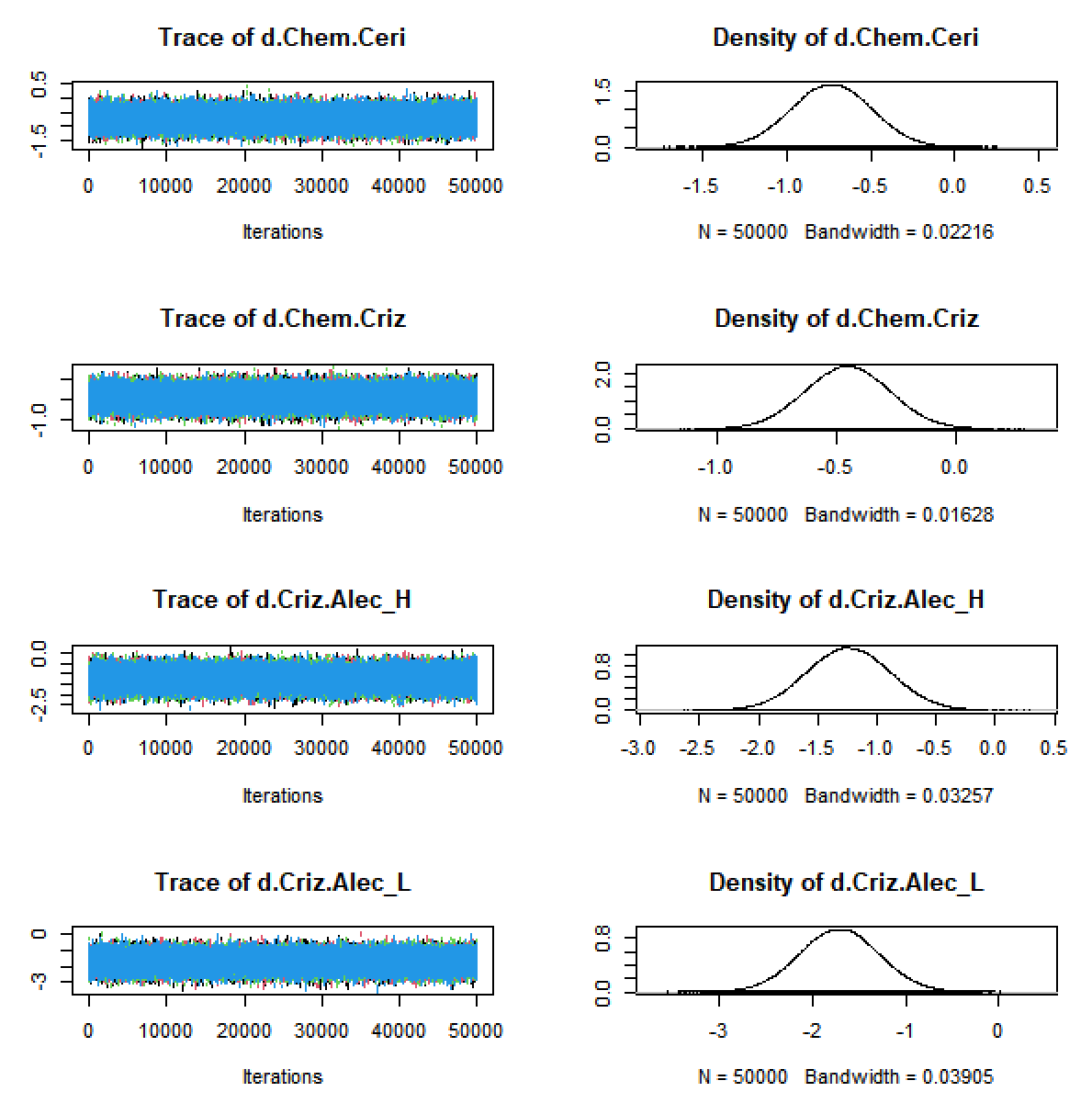

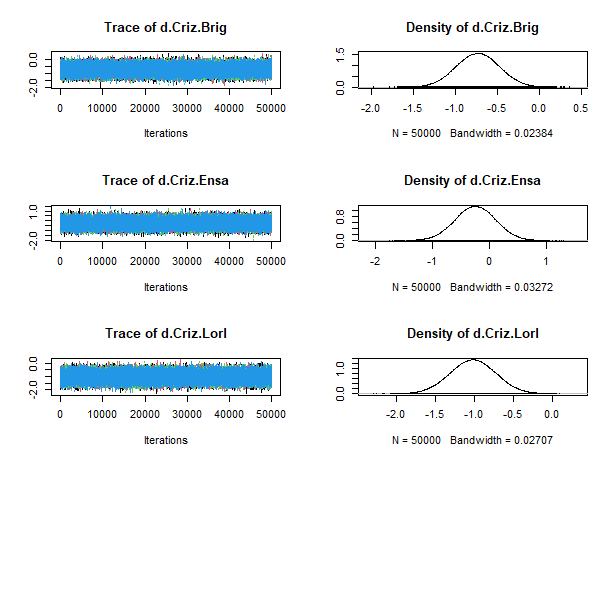


**(B)**


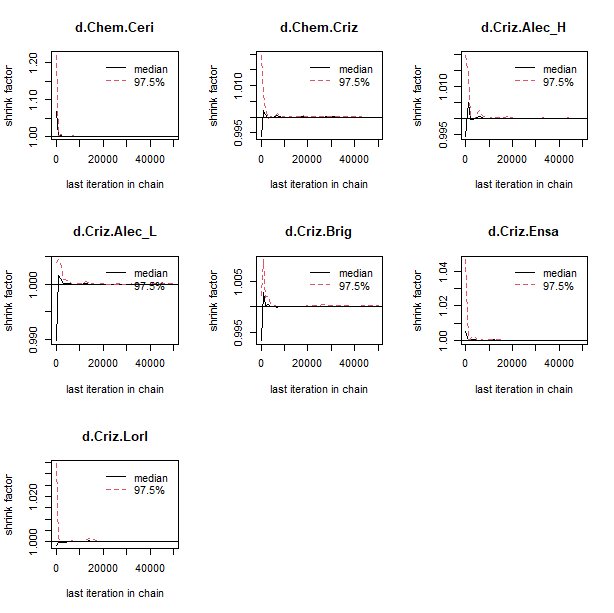

Supplement: Supplementary file 11 [file Table3.DOCX]

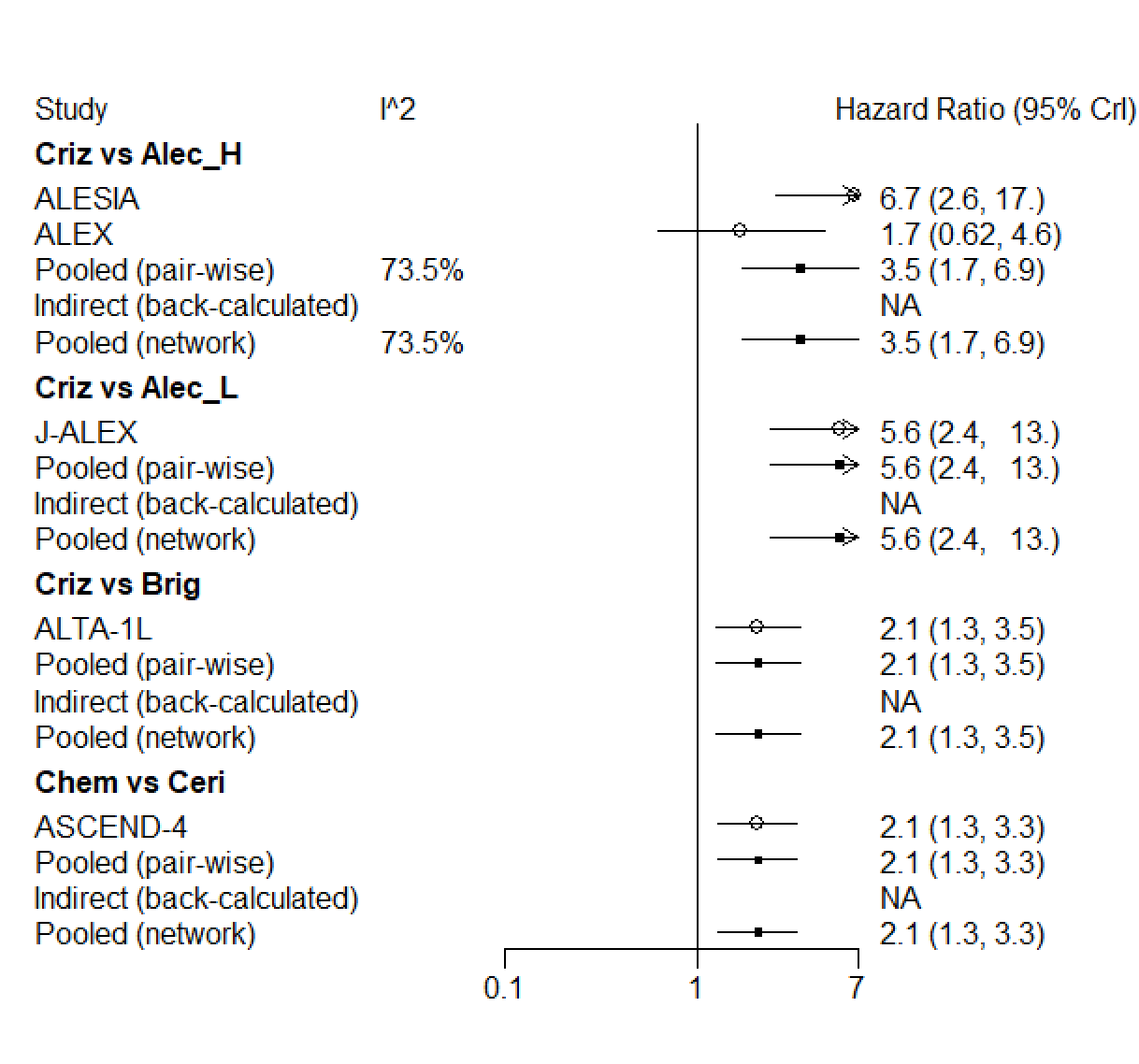

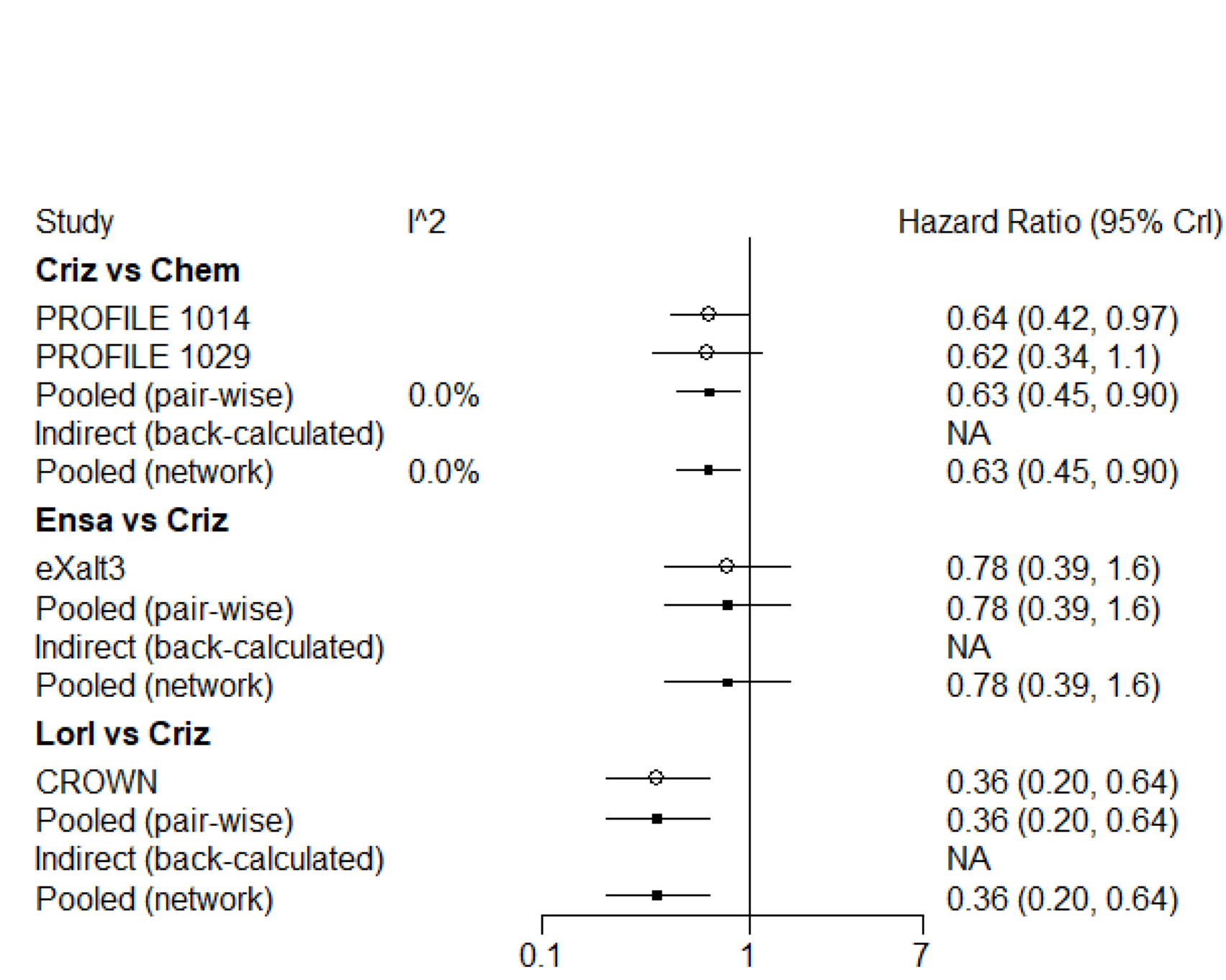

Supplement: Supplementary file 15 [file Table6.DOCX]

**(A)**


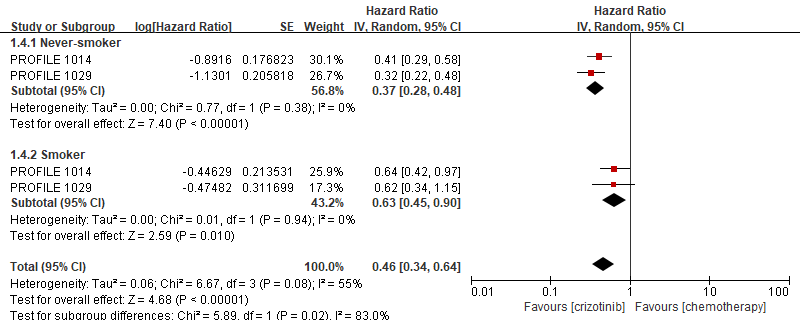


**(B)**


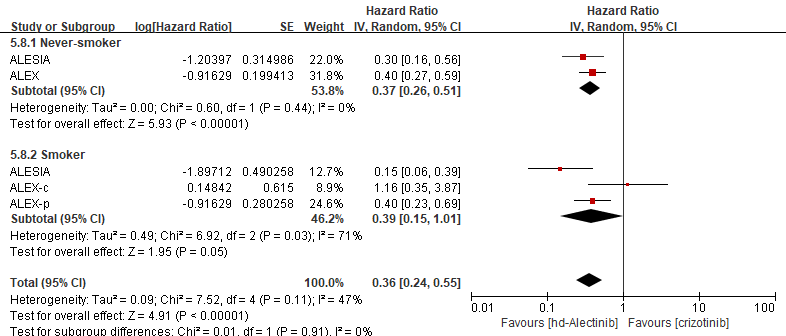

Supplement: Supplementary file 16 [file Table14.DOCX]
