# Supplementary material for: Impact of Smoking on Response to the First-Line Treatment of Advanced ALK-Positive Non-Small Cell Lung Cancer: A Bayesian Network Meta-Analysis
Source: Front Pharmacol. 2022 May 11;13:881493. doi: 10.3389/fphar.2022.881493 (PMC9130699; doi:10.3389/fphar.2022.881493)
Supplement: Supplementary file 2 [file Table15.DOCX]

| Supplementary Table 8 | | | | |
| --- | --- | --- | --- | --- |
| Group | Analysis | Model | DIC^1^ | DIC^2^ |
| Never-smoker group | Initial analysis | Fixed model | 15.33 | 15.39 |
|  |  | Random model | 16.67 | 16.80 |
|  | Sensitivity analysis | Fixed model | 9.38 | 9.35 |
|  |  | Random model | 10.67 | 10.73 |
| Smoker group | Initial analysis | Fixed model | 17.76 | 17.74 |
|  |  | Random model | 18.05 | 17.99 |
|  | Sensitivity analysis* | Fixed model | 11.76 | 11.76 |
|  |  | Random model | 12.01 | 11.97 |
|  | Sensitivity analysis! | Fixed model | 13.99 | 14.00 |
|  |  | Random model | 15.22 | 15.21 |
|  | Sensitivity analysis# | Fixed model | 7.99 | 8.00 |
|  |  | Random model | 9.06 | 9.05 |

| Supplementary Table 9 | | | | | | |  |
| --- | --- | --- | --- | --- | --- | --- | --- |
| Treatments | SUCRA Valus of nonsmoker | | SUCRA Valus of smoker | | | |  |
|  | Initial NMA | Sensitivity Analysis | Initial NMA | Sensitivity Analysis* | Sensitivity Analysis! | Sensitivity Analysis# | |
| Lorl | **89.53** | **93.61** | 66.75 | 75.24 | 72.09 | **88.02** | |
| Alec_L | 56.44 | NA | **85.86** | NA | **89.66** | NA | |
| Alec_H | 73.16 | 79.16 | 74.76 | **83.90** | 52.54 | 65.05 | |
| Brig | 63.11 | NA | 56.52 | NA | 61.89 | NA | |
| Ensa | 68.52 | NA | 38.60 | NA | 41.35 | NA | |
| Criz | 28.74 | 46.22 | 26.32 | 32.84 | 28.33 | 36.00 | |
| Ceri  Chem | 18.47  2.02 | 28.32  2.68 | 40.57  10.63 | 47.64  10.38 | 43.64  10.50 | 54.11  6.82 | |

| Supplementary Table 10 | | | | |
| --- | --- | --- | --- | --- |
| Trial | Experimental  drug | Control drug | HR1(95%CI) | HR2(95%CI) |
| ASCEND-4 | ceritinib | chemotherapy | 0.56(0.38-0.8) | 0.48(0.3-0.77) |
| J-ALEX | Ld-Alectinib | crizotinib | 0.5(0.28-0.89) | 0.18(0.08-0.42) |
| ALTA-1L | brigatinib | crizotinib | 0.43(0.28-0.65) | 0.48(0.29-0.8) |
| CROWN | lorlatinib | crizotinib | 0.24(0.14-0.4) | 0.36(0.2-0.63) |
| eXalt3 | ensartinib | crizotinib | 0.39(0.23-0.65) | 0.78(0.39-1.57) |
