# Supplementary material for: Impact of Smoking on Response to the First-Line Treatment of Advanced ALK-Positive Non-Small Cell Lung Cancer: A Bayesian Network Meta-Analysis
Source: Front Pharmacol. 2022 May 11;13:881493. doi: 10.3389/fphar.2022.881493 (PMC9130699; doi:10.3389/fphar.2022.881493)
Supplement: Supplementary file 3 [file Table12.DOCX]

| Treatments | SUCRA Valus of nonsmoker | | SUCRA Valus of smoker | | | |  |
| --- | --- | --- | --- | --- | --- | --- | --- |
|  | Initial NMA | Sensitivity Analysis | Initial NMA | Sensitivity Analysis* | Sensitivity Analysis! | Sensitivity Analysis# | |
| Lorl | **96.2** | **97.8** | 72.8 | 82.03 | 79.6 | 94.06 | |
| Alec_L | 54.5 | NA | **95.5** | NA | **97.8** | NA | |
| Alec_H | 74.1 | 77.2 | 81.6 | **91.55** | 53.6 | 67.04 | |
| Brig | 62.5 | NA | 58.8 | NA | 66.2 | NA | |
| Ensa | 69.7 | NA | 34.3 | NA | 38.7 | NA | |
| Criz | 28.2 | 49.1 | 20.2 | 29.30 | 22.3 | 32.97 | |
| Ceri  Chem | 14.8  0.00 | 25.9  0.00 | 36.2  0.01 | 46.99  0.14 | 40.7  0.11 | 54.93  1.00 | |
