# Supplementary material for: Impact of Smoking on Response to the First-Line Treatment of Advanced ALK-Positive Non-Small Cell Lung Cancer: A Bayesian Network Meta-Analysis
Source: Front Pharmacol. 2022 May 11;13:881493. doi: 10.3389/fphar.2022.881493 (PMC9130699; doi:10.3389/fphar.2022.881493)
Supplement: Supplementary file 7 [file Table9.DOCX]

| **Certainty assessment** | | | | | | | **№ of patients** | | **Effect** | | **Certainty** | **Importance** |
| --- | --- | --- | --- | --- | --- | --- | --- | --- | --- | --- | --- | --- |
| **№ of studies** | **Study design** | **Risk of bias** | **Inconsistency** | **Indirectness** | **Imprecision** | **Other considerations** | **TKI** | **Chem** | **Relative (95% CI)** | **Absolute (95% CI)** |  |  |
| **ALK-TKI vs Chem - nonsmoker** | | | | | | | | | | | | |
| 3 | randomised trials | serious^a^ | not serious | not serious | not serious | none | 289 participants | 306 participants | **HR 0.42** (0.31 to 0.57) [Disease progression or death] | **-- per 1,000** (from -- to --) | ⨁⨁⨁◯ Moderate | CRITICAL |
|  |  |  |  |  |  |  | - | 0.0% |  | **-- per 1,000** (from -- to --) |  |  |
| **ALK-TKI vs Chem - smoker** | | | | | | | | | | | | |
| 3 | randomised trials | serious^a^ | not serious | not serious | serious^b^ | none |  |  | **HR 0.58** (0.44 to 0.76) [Disease progression or death] | **-- per 1,000** (from -- to --) | ⨁⨁◯◯ Low | CRITICAL |
|  |  |  |  |  |  |  | - | 0.0% |  | **-- per 1,000** (from -- to --) |  |  |
