# Supplementary material for: Impact of Smoking on Response to the First-Line Treatment of Advanced ALK-Positive Non-Small Cell Lung Cancer: A Bayesian Network Meta-Analysis
Source: Front Pharmacol. 2022 May 11;13:881493. doi: 10.3389/fphar.2022.881493 (PMC9130699; doi:10.3389/fphar.2022.881493)
Supplement: Supplementary file 12 [file Table13.DOCX]

|  | [,1] | [,2] | [,3] | [,4] | [,5] | [,6] | [,7] | [,8] |
| --- | --- | --- | --- | --- | --- | --- | --- | --- |
| Alec_H | 0.174575 | 0.490105 | 0.230780 | 0.082795 | 0.018250 | 0.003255 | 0.000215 | 0.000025 |
| Alec_L | 0.769475 | 0.165475 | 0.049055 | 0.013290 | 0.002270 | 0.000400 | 0.000035 | 0.000000 |
| Brig | 0.005050 | 0.059890 | 0.220155 | 0.516695 | 0.160110 | 0.036465 | 0.001600 | 0.000035 |
| Ceri | 0.000240 | 0.003625 | 0.022300 | 0.102160 | 0.401040 | 0.320795 | 0.148700 | 0.001140 |
| Chem | 0.000000 | 0.000000 | 0.000000 | 0.000000 | 0.000040 | 0.001525 | 0.041410 | 0.957025 |
| Criz | 0.000000 | 0.000000 | 0.000000 | 0.000110 | 0.042650 | 0.332870 | 0.620475 | 0.003895 |
| Ensa | 0.000570 | 0.006900 | 0.029650 | 0.103625 | 0.335890 | 0.298105 | 0.187390 | 0.037870 |
| Lorl | 0.050090 | 0.274005 | 0.448060 | 0.181325 | 0.039750 | 0.006585 | 0.000175 | 0.000010 |
