# Supplementary material for: Impact of Smoking on Response to the First-Line Treatment of Advanced ALK-Positive Non-Small Cell Lung Cancer: A Bayesian Network Meta-Analysis
Source: Front Pharmacol. 2022 May 11;13:881493. doi: 10.3389/fphar.2022.881493 (PMC9130699; doi:10.3389/fphar.2022.881493)
Supplement: Supplementary file 13 [file Table4.DOCX]

**(A)** **(B)**

**(C)** **(D)**

**(E)** **(F)**

**(G)** **(H)**

**(I)** **(J)**
