# Supplementary material for: Impact of Smoking on Response to the First-Line Treatment of Advanced ALK-Positive Non-Small Cell Lung Cancer: A Bayesian Network Meta-Analysis
Source: Front Pharmacol. 2022 May 11;13:881493. doi: 10.3389/fphar.2022.881493 (PMC9130699; doi:10.3389/fphar.2022.881493)
Supplement: Supplementary file 14 [file Table11.DOCX]

|  | [,1] | [,2] | [,3] | [,4] | [,5] | [,6] | [,7] | [,8] |
| --- | --- | --- | --- | --- | --- | --- | --- | --- |
| Alec_H | 0.057750 | 0.368485 | 0.333760 | 0.184995 | 0.055010 | 0.000000 | 0.000000 | 0.000000 |
| Alec_L | 0.019615 | 0.088120 | 0.130525 | 0.218590 | 0.533150 | 0.009070 | 0.000930 | 0.000000 |
| Brig | 0.023820 | 0.149000 | 0.244855 | 0.344275 | 0.237975 | 0.000075 | 0.000000 | 0.000000 |
| Ceri | 0.000000 | 0.000000 | 0.000000 | 0.000025 | 0.001005 | 0.036580 | 0.961190 | 0.001200 |
| Chem | 0.000000 | 0.000000 | 0.000000 | 0.000000 | 0.000000 | 0.000000 | 0.001200 | 0.998800 |
| Criz | 0.000000 | 0.000000 | 0.000000 | 0.000000 | 0.009275 | 0.954060 | 0.036665 | 0.000000 |
| Ensa | 0.077035 | 0.277565 | 0.250105 | 0.236005 | 0.159060 | 0.000215 | 0.000015 | 0.000000 |
| Lorl | 0.821780 | 0.116830 | 0.040755 | 0.016110 | 0.004525 | 0.000000 | 0.000000 | 0.000000 |
