# Supplementary material for: Impact of Smoking on Response to the First-Line Treatment of Advanced ALK-Positive Non-Small Cell Lung Cancer: A Bayesian Network Meta-Analysis
Source: Front Pharmacol. 2022 May 11;13:881493. doi: 10.3389/fphar.2022.881493 (PMC9130699; doi:10.3389/fphar.2022.881493)
Supplement: Supplementary file 18 [file Table10.DOCX]

| **Certainty assessment** | | | | | | | **№ of patients** | | **Effect** | | **Certainty** | **Importance** |
| --- | --- | --- | --- | --- | --- | --- | --- | --- | --- | --- | --- | --- |
| **№ of studies** | **Study design** | **Risk of bias** | **Inconsistency** | **Indirectness** | **Imprecision** | **Other considerations** | **2/3G TKI** | **1G TKI** | **Relative (95% CI)** | **Absolute (95% CI)** |  |  |
| **2/3 G ALK-TKI vs 1G ALK-TKI - nonsmoker** | | | | | | | | | | | | |
| 6 | randomised trials | serious^a^ | not serious | not serious | not serious | none | 471 participants | 456 participants | **HR 0.37** (0.31 to 0.46) [Disease progression or death] | **-- per 1,000** (from -- to --) | ⨁⨁⨁◯ Moderate | CRITICAL |
|  |  |  |  |  |  |  | - | 0.0% |  | **-- per 1,000** (from -- to --) |  |  |
| **2/3 G** **ALK-TKI vs 1G ALK-TKI - smoker** | | | | | | | | | | | | |
| 6 | randomised trials | serious^a^ | serious^b^ | not serious | serious^c^ | none | 316 participants | 271 participants | **HR 0.40** (0.26 to 0.60) [Disease progression or death] | **-- per 1,000** (from -- to --) | ⨁◯◯◯ Very low | CRITICAL |
|  |  |  |  |  |  |  | - | 0.0% |  | **-- per 1,000** (from -- to --) |  |  |
| **2/3 G ALK-TKI vs 1G ALK-TKI-multiracial subgroup - nonsmoker** | | | | | | | | | | | | |
| 4 | randomised trials | serious^a^ | not serious | not serious | not serious | none | 331 participants | 350 participants | **HR 0.37** (0.29 to 0.47) [Disease progression or death] | **-- per 1,000** (from -- to --) | ⨁⨁⨁◯ Moderate | IMPORTANT |
|  |  |  |  |  |  |  | - | 0.0% |  | **-- per 1,000** (from -- to --) |  |  |
| **2/3 G ALK-TKI vs 1G ALK-TKI-multiracial subgroup - smoker** | | | | | | | | | | | | |
| 4 | randomised trials | serious^a^ | not serious | not serious | not serious | none | 228 participants | 211 participants | **HR 0.50** (0.36 to 0.69) [Disease progression or death] | **-- per 1,000** (from -- to --) | ⨁⨁⨁◯ Moderate | IMPORTANT |
|  |  |  |  |  |  |  | - | 0.0% |  | **-- per 1,000** (from -- to --) |  |  |
| **2/3 G ALK-TKI vs 1G** **ALK- TKI-Asian-only subgroup- nonsmoker** | | | | | | | | | | | | |
| 2 | randomised trials | serious^a^ | not serious | not serious | not serious | none | 140 participants | 106 participants | **HR 0.39** (0.24 to 0.65) [Disease progression or death] | **-- per 1,000** (from -- to --) | ⨁⨁⨁◯ Moderate | IMPORTANT |
|  |  |  |  |  |  |  | - | 0.0% |  | **-- per 1,000** (from -- to --) |  |  |
| **2/3 G** **ALK- TKI vs 1G** **ALK- TKI-Asian-only subgroup- smoker** | | | | | | | | | | | | |
| 2 | randomised trials | serious^a^ | not serious | not serious | serious^c^ | none | 88 participants | 60 participants | **HR 0.17** (0.09 to 0.31) [Disease progression or death] | **-- per 1,000** (from -- to --) | ⨁⨁◯◯  Low | IMPORTANT |
|  |  |  |  |  |  |  | - | 0.0% |  | **-- per 1,000** (from -- to --) |  |  |
